# Global and regional burden of first-ever ischaemic and haemorrhagic stroke during 1990–2010: findings from the Global Burden of Disease Study 2010

**DOI:** 10.1016/S2214-109X(13)70089-5

**Published:** 2013-10-24

**Authors:** Rita V Krishnamurthi, Valery L Feigin, Mohammad H Forouzanfar, George A Mensah, Myles Connor, Derrick A Bennett, Andrew E Moran, Ralph L Sacco, Laurie M Anderson, Thomas Truelsen, Martin O’Donnell, Narayanaswamy Venketasubramanian, Suzanne Barker-Collo, Carlene M M Lawes, Wenzhi Wang, Yukito Shinohara, Emma Witt, Majid Ezzati, Mohsen Naghavi, Christopher Murray

**Affiliations:** National Institute for Stroke and Applied Neurosciences, Faculty of Health and Environmental Studies, Auckland University of Technology, Auckland, New Zealand (R V Krishnamurthi PhD, Prof V L Feigin MD, E Witt MSc); Institute for Health Metrics and Evaluation, Department of Global Health, University of Washington, Seattle, WA, USA (Prof M H Forouzanfar MD, Prof M Naghavi MD, Prof C Murray MD); National Institutes of Health Heart, Lung, and Blood Institute, Bethesda, MD, USA (G A Mensah, MD); Consultant Neurologist, National Health Service Borders, Melrose, UK (M Connor MBBCh); Division of Clinical Neurosciences, University of Edinburgh, UK (M Connor MBBCh); Bute Medical School, University of St Andrews, UK (M Connor); School of Public Health, University of the Witwatersrand, South Africa (M Connor); Clinical Trial Service Unit and Epidemiological Studies Unit, Nuffield Department of Population Health, University of Oxford, Oxford, UK (D A Bennett PhD); Division of General Medicine, Columbia University Medical Center, NY, USA (Prof A E Moran MD); Miller School of Medicine, University of Miami, Miami, FL, USA (Prof R L Sacco MD); Department of Epidemiology, School of Public Health, University of Washington, and Washington State Institute for Public Policy, WA, USA (L M Anderson PhD); Department of Neurology, Copenhagen University Hospital Herlev, Herlev, Denmark (T Truelsen MD); MRC-HPA Centre for Environment and Health, Department of Epidemiology and Biostatistics, School of Public Health, Imperial College London, London, UK (Prof M Ezzati PhD); National University of Ireland, Galway, Ireland (Prof M O’Donnell PhD); Division of Neurology, University Medicine Cluster, Yong Loo Lin School of Medicine, and Saw Swee Hock School of Public Health, National University of Singapore, Singapore (N Venketasubramanian MBBS); School of Psychology (Prof S Barker-Collo PhD) and National Institute for Health Innovation (C M M Lawes MBChB), University of Auckland, Auckland, New Zealand; Beijing Neurosurgical Institute, Beijing, China (W Wang PhD); and Federation of National Public Service Personnel Mutual Aid Associations Tachikawa Hospital, Tachikawa, Tokyo, Japan (Y Shinohara, MD)

## Abstract

**Background:**

The burden of ischaemic and haemorrhagic stroke varies between regions and over time. With differences in prognosis, prevalence of risk factors, and treatment strategies, knowledge of stroke pathological type is important for targeted region-specific health-care planning for stroke and could inform priorities for type-specific prevention strategies. We used data from the Global Burden of Diseases, Injuries, and Risk Factors Study 2010 (GBD 2010) to estimate the global and regional burden of first-ever ischaemic and haemorrhagic stroke during 1990–2010.

**Methods:**

We searched Medline, Embase, LILACS, Scopus, PubMed, Science Direct, Global Health Database, the WHO library, and regional databases from 1990 to 2012 to identify relevant studies published between 1990 and 2010. We applied the GBD 2010 analytical technique (DisMod-MR) to calculate regional and country-specific estimates for ischaemic and haemorrhagic stroke incidence, mortality, mortality-to-incidence ratio, and disability-adjusted life-years (DALYs) lost, by age group (aged <75 years, ≥75 years, and in total) and country income level (high-income and low-income and middle-income) for 1990, 2005, and 2010.

**Findings:**

We included 119 studies (58 from high-income countries and 61 from low-income and middle-income countries). Worldwide, the burden of ischaemic and haemorrhagic stroke increased significantly between 1990 and 2010 in terms of the absolute number of people with incident ischaemic and haemorrhagic stroke (37% and 47% increase, respectively), number of deaths (21% and 20% increase), and DALYs lost (18% and 14% increase). In the past two decades in high-income countries, incidence of ischaemic stroke reduced significantly by 13% (95% CI 6–18), mortality by 37% (19–39), DALYs lost by 34% (16–36), and mortality-to-incidence ratios by 21% (10–27). For haemorrhagic stroke, incidence reduced significantly by 19% (1–15), mortality by 38% (32–43), DALYs lost by 39% (32–44), and mortality-to-incidence ratios by 27% (19–35). By contrast, in low-income and middle-income countries, we noted a significant increase of 22% (5–30) in incidence of haemorrhagic stroke and a 6% (–7 to 18) non-significant increase in the incidence of ischaemic stroke. Mortality rates for ischaemic stroke fell by 14% (9–19), DALYs lost by 17% (–11 to 21%), and mortality-to-incidence ratios by 16% (–12 to 22). For haemorrhagic stroke in low-income and middle-income countries, mortality rates reduced by 23% (–18 to 25%), DALYs lost by 25% (–21 to 28), and mortality-to-incidence ratios by 36% (–34 to 28).

**Interpretation:**

Although age-standardised mortality rates for ischaemic and haemorrhagic stroke have decreased in the past two decades, the absolute number of people who have these stroke types annually, and the number with related deaths and DALYs lost, is increasing, with most of the burden in low-income and middle-income countries. Further study is needed in these countries to identify which subgroups of the population are at greatest risk and who could be targeted for preventive efforts.

## Introduction

Investigation of stroke burden by its major pathological types, and study of their secular trends in different regions of the world, is important for targeted region-specific health-care planning in stroke (eg, estimation of resources needed to care for patients with stroke, by type) and can inform priorities for type-specific prevention strategies. These data are also important for improving understanding of the health consequences and patterns of epidemiological transitions reported worldwide. Findings from systematic reviews suggest that low-income and middle-income countries have a greater proportion of haemorrhagic stroke than do high-income countries,^1^ that geographical variation is high in the incidence of major pathological types of stroke,^1^ and that no substantial changes have taken place in the incidence of haemorrhagic stroke in the past three decades.^2,3^ However, no detailed and systematic comprehensive estimates have been made of the global and regional incidence, case-fatality, disability-adjusted life-years (DALYs) lost, and secular trends of incidence of ischaemic or haemorrhagic stroke, especially for low-income and middle-income countries.^4–6^ We report estimates from the Global Burden of Diseases, Injuries, and Risk Factors Study (GBD 2010) for incidence, mortality, mortality-to-incidence ratio, and DALYs lost in ischaemic or haemorrhagic stroke in all 21 regions of the world^7^ in 1990, 2005, and 2010.

## Methods

### Systematic literature review

We did a systematic literature review to establish the review process. The search strategy and selection criteria and have been described elsewhere.^8,9^ We assessed pathological types of stroke for studies that used head CT or MRI within the first 2 weeks of stroke onset, or for those in which brain autopsy findings for confirmation of type were available for at least 70% of stroke cases. We categorised pathological stroke types into two groups—ischaemic and haemorrhagic (intracerebral haemorrhage and subarachnoid haemorrhage combined). We included all age groups in the analysis. We analysed only first-ever stroke events.

### Calculation of incidence, mortality, and DALYs lost

We have described our statistical analysis strategies elsewhere and in a companion report in *The Lancet.*^8,9^ Briefly, we applied the GBD 2010 analytical technique (DisMod-MR) to calculate regional and country-specific estimates of incidence and mortality per 100 000 person-years for ischaemic and haemorrhagic stroke, and of DALYs lost per 100 000 people, by age group (<20 years, 20–64 years, 65–74 years, ≤75 years, total) and level of country income (high and low and middle) for 1990, 2005, and 2010.

### Disease modelling

For modelling of mortality for ischaemic and haemorrhagic stroke, we selected a set of relevant covariates (appendix) and assumed a plausible direction of effect on the basis of existing published work. The ensemble approach combined different model results developed with different combinations of covariates and statistical approaches.^10^ In a separate process—the CODCorrect process^11^—the number of deaths from ischaemic or haemorrhagic stroke were rescaled to total the overall number of deaths (mortality envelope) for a country, sex, and age group. This process also ensured that the sum of cardiovascular disease deaths was equal to all deaths from cardiovascular disease (modelled independently). The appendix shows the estimates before and after correction for stroke deaths. We report age-standardised incidence and mortality rates per 100 000 person-years and estimates of DALYs lost per 100 000 people with the direct method of standardisation and WHO’s standard population as a reference.^12^ Additionally, we calculated mortality-to-incidence ratio for each region and country as an indicator of the success or failure of stroke management strategies in a particular region (ratio numbers were based on the total number of incident cases and deaths). We estimated p values on the basis of 1000 draws of the posterior distribution of each statistic. Because some posterior distributions deviated significantly from normal, 2·5 and 97·5 percentiles of the draws were reported as the lower and upper bounds of the uncertainty interval for the statistic. We calculated 95% CIs for all rates.

### Role of the funding source

The sponsor of the study had no role in study design, data collection, data analysis, data interpretation, or writing of the report. The Writing and GBD Global Analysis Group had full access to all the data in the study and had final responsibility for the decision to submit for publication.

## Results

Characteristics of studies included in the analysis are described in the accompanying *Lancet* paper.^9^ We included 119 studies (58 from high-income countries and 61 from low-income and middle-income countries) in this analysis.

Worldwide in 2010, an estimated 11 569 538 events of incident ischaemic stroke took place (63% in low-income and middle-income countries), and 5 324 997 events of incident haemorrhagic stroke (80% in low-income and middle-income countries); furthermore, 2 835 419 individuals died from ischaemic stroke (57% in low-income and middle-income countries) and 3 038 763 from haemorrhagic stroke (84% in low-income and middle-income countries; appendix). In 2010, 39 389 408 DALYs were lost because of ischaemic stroke (64% in low-income and middle-income countries) and 62 842 896 because of haemorrhagic stroke (86% in low-income and middle-income countries; appendix). In 2010, age-standardised incidence per 100 000 person-years of ischaemic stroke ranged from 51·88 in Qatar to 433·97 in Lithuania (table 1); incidence of haemorrhagic stroke ranged from 14·55 in Qatar to 159·81 in China; (table 2). Age-standardised mortality rates per 100 000 person-years for ischaemic stroke ranged from 9·17 in Qatar to 137·70 in Russia (table 1); the rate of haemorrhagic stroke ranged from 9·64 in the USA to 210·56 in Mongolia (table 2). DALYs lost per 100 000 people because of ischaemic stroke ranged from 163·89 in Israel to 2032·11 in Afghanistan (table 1); for haemorrhagic stroke the number of DALYs lost ranged from 178·20 in Switzerland to 4118·90 in Mongolia (table 2).

In the past two decades in high-income countries, incidence of ischaemic stroke significantly reduced by 13% (95% CI 6–18), mortality by 37% (19–39), DALYs by 34% (16–36), and mortality-to-incidence ratios by 21% (10–27; table 3). Reductions shown for haemorrhagic stroke were 19% (1–15%) for incidence, 38% (32–43%) for mortality, 39% (32–44%) for DALYs, and 27% (19–35%) for mortality-to-incidence ratio (table 3). Reductions in incidence in both stroke groups were significant for the younger age group (<75 years, from 110·80/100 000 [95% CI 103·05–118·54] to 100·47/100 000 [94·03–107·16], p=0·021, for incidence of ischaemic stroke, and from 41·92/100 000 [38·89–45·15] to 38·46/100 000 [35·68–41·16], p=0·038, for incidence of haemorrhagic stroke). Worldwide, in the younger age group, the incidence of ischaemic stroke did not change, but we noted a significant increase in the incidence of haemorrhagic stroke, from 54·07 (48·56–60·22) to 64·07 (56·45–73·33; p=0·028). In the older age group (≥75 years) we noted no significant change in the incidence of ischaemic stroke (from 2614·89/100 000 [2426·49–2809·55] to 2472·93/100 000 [2279·15–2687·39], p=0·176), whereas a significant reduction was shown in the incidence of haemorrhagic stroke (from 558·61/100 000 [503·36–624·07] to 640·06/100 000 [569·10–724·72], p=0·046).

We noted a significant increase of 22% (95% CI 5–30) in the incidence of haemorrhagic stroke in low-income and middle-income countries in the past two decades, with a 19% (5–30) significant increase in people younger than 75 years. A non-significant increase of 6% (18%, –7 to 32) was shown in the incidence of ischaemic stroke; additionally, mortality rates were reduced by 14% (–2% to 32), DALYs lost by 16% (1–35%), and mortality-to-incidence ratio by 16% (–5% to 37); however, these differences were not significant. Similarly for haemorrhagic stroke, mortality rates were reduced by 23% (–3% to 36%), DALYs lost by 25% (7–38%), and mortality-to-incidence ratio by 36% (16–49%), likewise not significantly. In the past two decades, the incidence of both ischaemic and haemorrhagic stroke in low- income and middle-income countries increased signi-ficantly in people aged 20–64 years (table 3). Worldwide, the mean age of people with incident and fatal stroke has increased in the past two decades, with the largest increase noted in high-income countries (table 4). In 2010, the mean age of patients with incident and fatal ischaemic and haemorrhagic stroke was 3–5 years greater in high-income than in low-income to middle-income countries (table 4).

**Figure 1:**
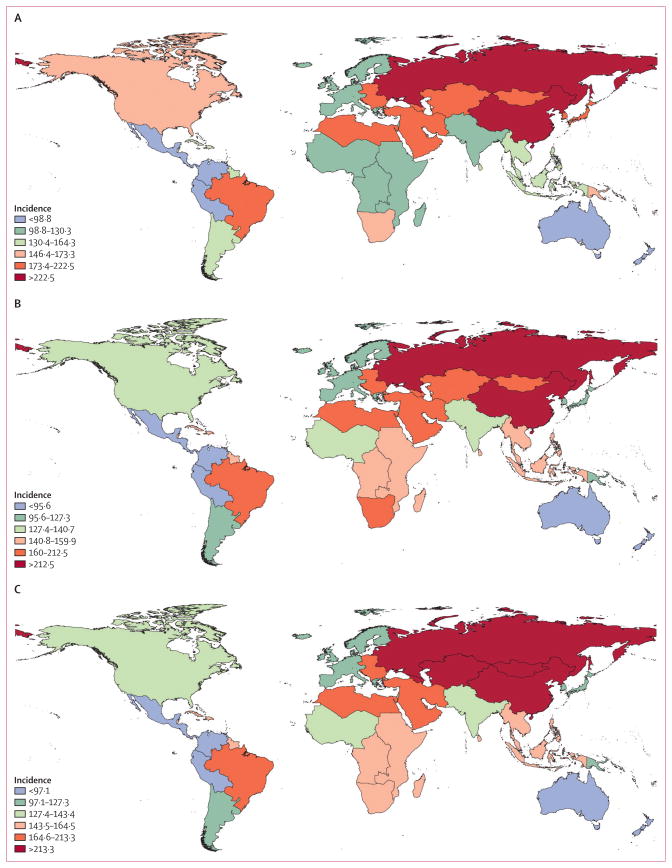
Age-standardised incidence of ischaemic stroke per 100 000 person-years for 1990 (A), 2005 (B), and 2010 (C)

**Figure 2:**
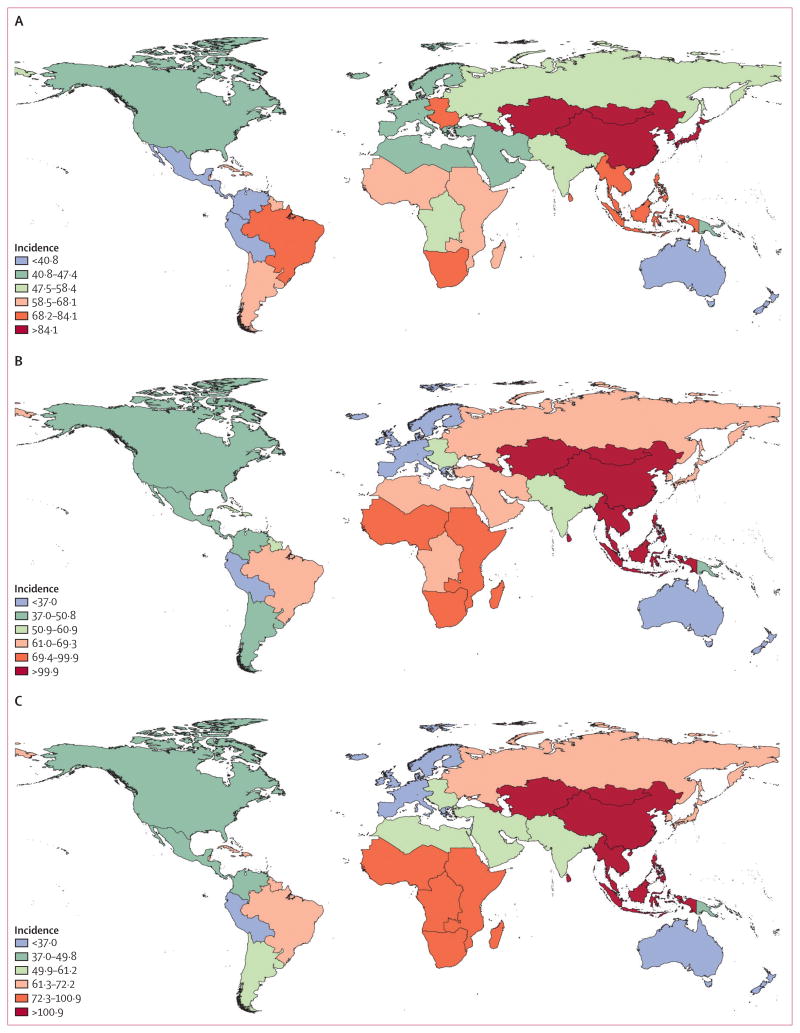
Age-standardised incidence of haemorrhagic stroke per 100 000 person-years for 1990 (A), 2005 (B), and 2010 (C)

**Figure 3:**
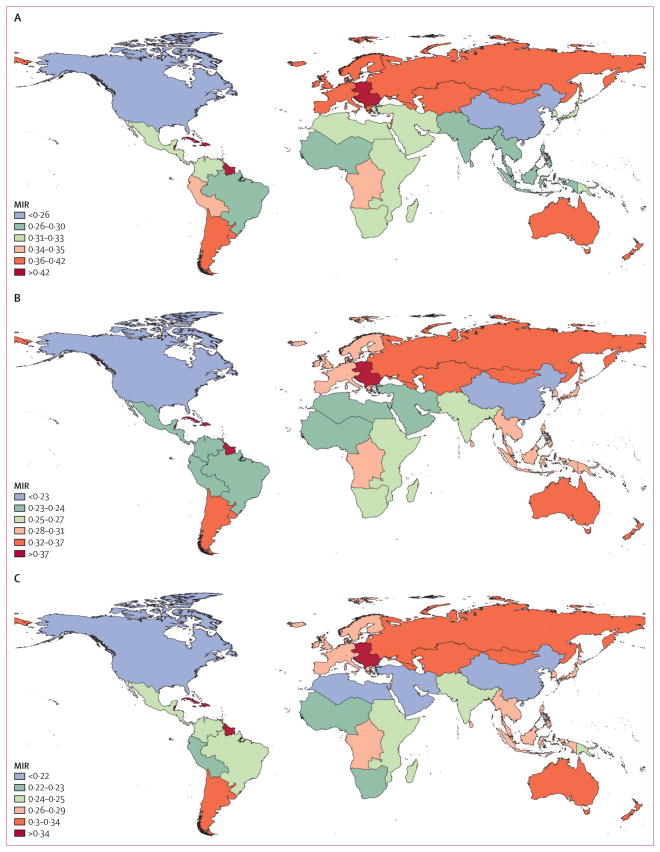
Mortality-to-incidence ratio (MIR) for ischaemic stroke for 1990 (A), 2005 (B), and 2010 (C)

**Figure 4:**
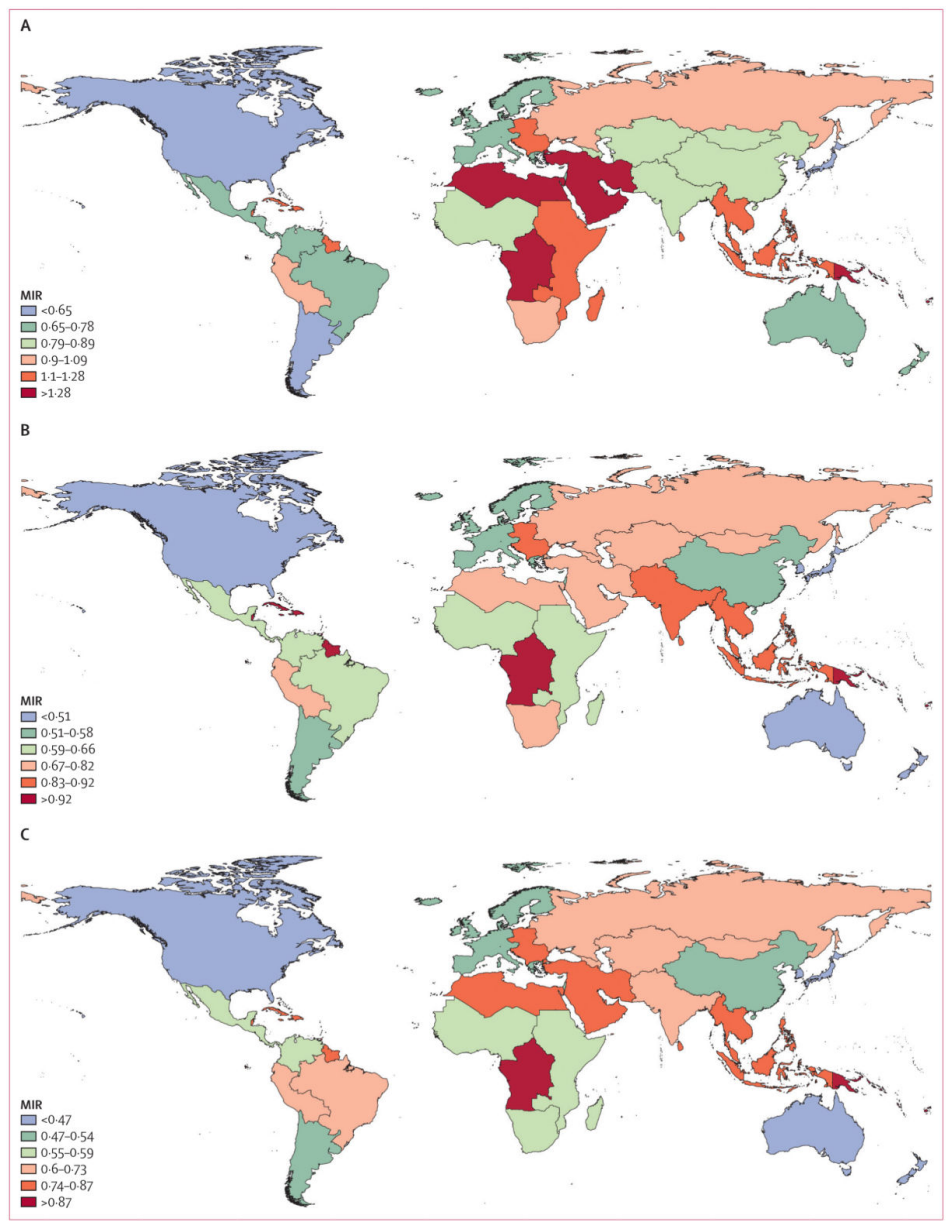
Mortality-to-incidence ratio (MIR) for haemorrhagic stroke for 1990 (A), 2005 (B), and 2010 (C)

By GBD region, in the past two decades, the largest increases in incidence of ischaemic stroke were in eastern Europe, central and east Asia, north and sub- Saharan Africa, and the Middle East (figure 1), with the largest increase (22%) noted in the Democratic Republic of Congo. Notably, some of the largest decreases in incidence of ischaemic stroke between 1990 and 2010 were also in these regions (South Korea 44%, Chile 41%, Brunei 41%; figure 1). Up to 2010, the highest rates of ischaemic stroke were in eastern Europe (particularly Russia: 238–416/100 000) and central and east Asia, North Africa, and the Middle East (178–238/100 000). The largest increases in incidence of haemorrhagic stroke by GBD region were in eastern and central Europe, North and sub-Saharan Africa, and the Middle East, whereas in high-income regions of North America, western Europe, and tropical and southern Latin America incidence of haemorrhagic stroke decreased significantly (figure 2). In 2010, the highest incidences of haemorrhagic stroke were in central and east Asia (101–158/100 000) and east and southern sub-Sahara Africa (73–101/100 000), whereas the lowest rates were in high-income North America, central and Andean Latin America, western Europe, and Oceania (Australasia; 25–40/100 000). Between 1990 and 2010, mortality-to-incidence ratios for ischaemic stroke noticeably reduced in western Europe, Australasia, and central and Andean Latin America, but increased in North Africa, the Middle East, and southeast Asia (figure 3). For haemorrhagic stroke, we noted decreases in mortality-to-incidence ratios in northern Africa; the Middle East; central, east, and southern sub-Saharan Africa; and east and southeast Asia, whereas moderate increases were evident in central Latin America and high-income Asia-Pacific regions (figure 4).

**Figure 5:**
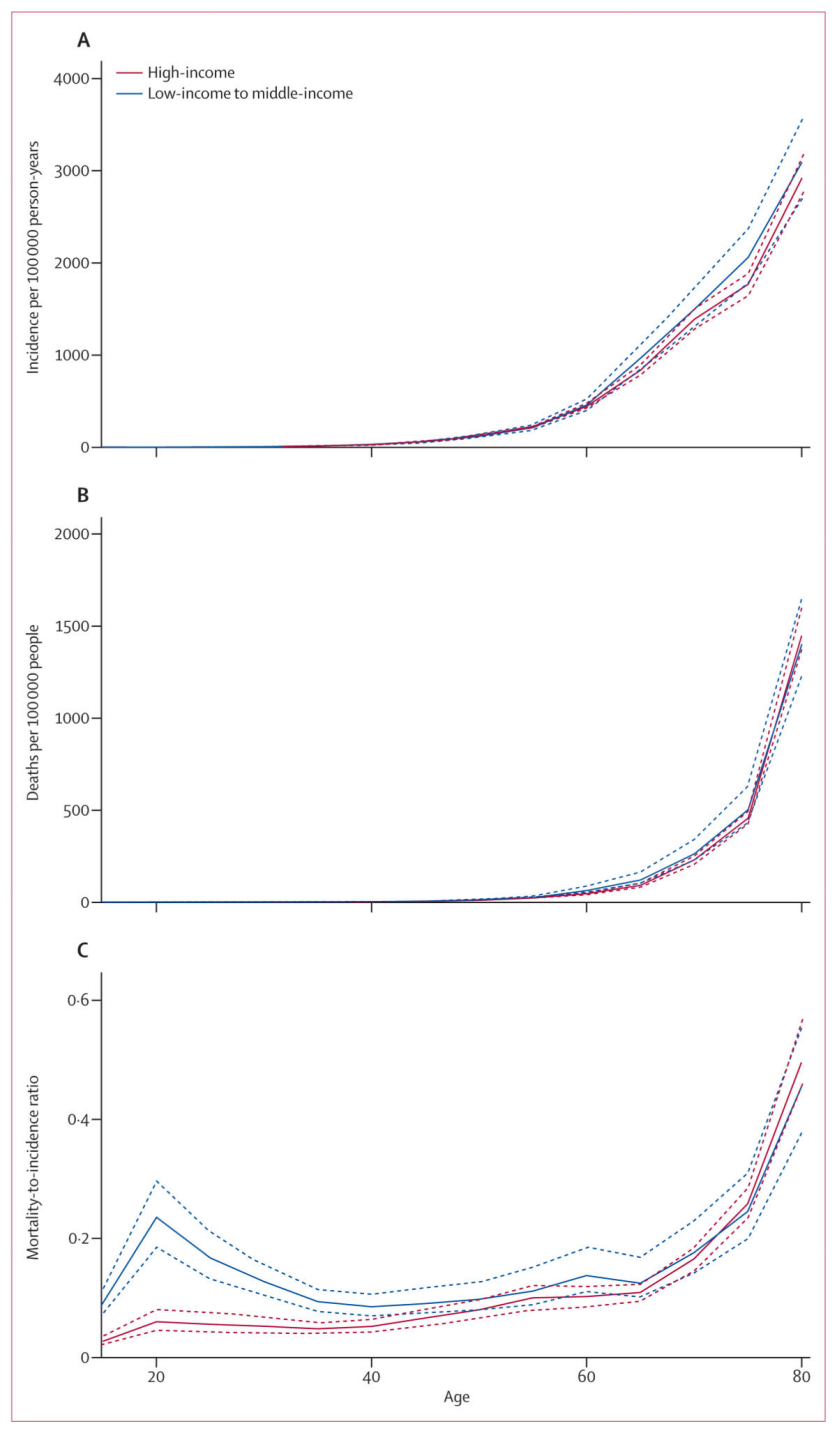
Incidence (A), mortality (B), and mortality-to-incidence ratio (C) for ischaemic stroke, by age and country income level, for 2010

**Figure 6:**
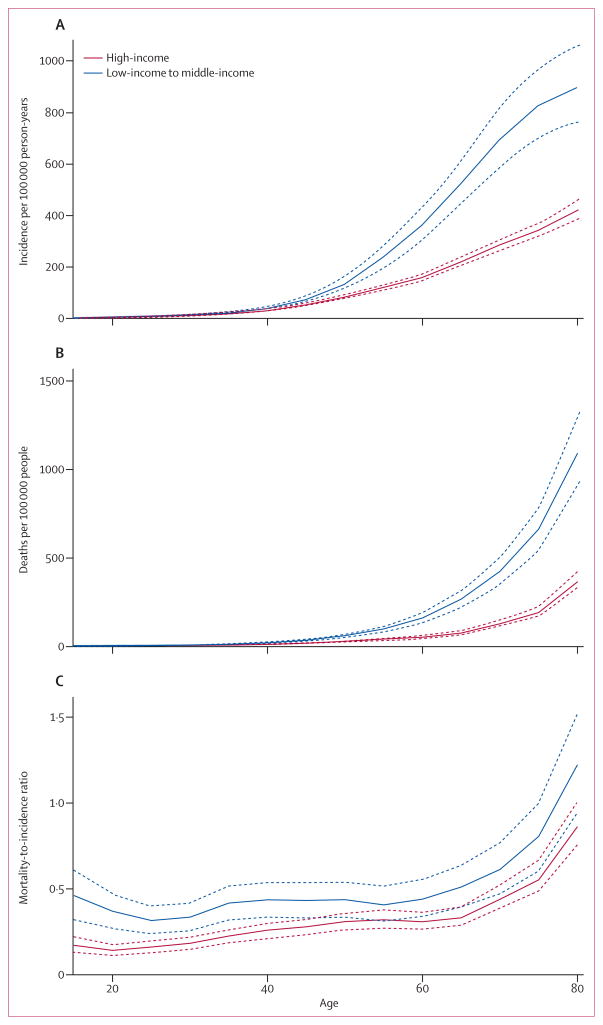
Incidence (A), mortality (B), and mortality-to-incidence ratio (C) for haemorrhagic stroke, by age and country income level, for 2010

In 2010, the lowest mortality-to-incidence ratios for ischaemic stroke were in high-income North America and east Asia (0·17–0·19) and for haemorrhagic stroke in high-income North America (0·25). The highest mortality-to-incidence ratios for ischaemic stroke were in central Europe and the Caribbean (0·34–0·38), and for haemorrhagic stroke in Oceania (0·94–1·27). In 2010, the age-specific incidences of ischaemic and haemorrhagic stroke increased significantly with age in all GBD regions (figures 5, 6). Although we noted no differences in the age-specific incidence of ischaemic stroke between high-income and low-income countries (figure 5), age-specific rates of haemorrhagic stroke increased in low-income to middle-income countries (figure 6). Age-specific rates were significantly greater in people older than 45 years in low-income to middle-income countries than in high-income countries. Age-specific mortality rates, mortality-to-incidence ratios, and DALYs for both stroke types were greater overall in low-income to middle-income countries than in high-income countries, but significant differences between higher-income and lower-income countries were only apparent for haemorrhagic stroke incidence, mortality, DALYs in people older than 40 years, and for mortality-to-incidence ratios across all age groups (figures 5, 6).

## Discussion

This study is the first to report the global burden of ischaemic and haemorrhagic stroke in terms of incidence, mortality, DALYs lost, and mortality-to-incidence ratio across GBD regions and countries in 1990, 2005, and 2010, and across all age groups of the population. Several important findings were shown (panel). First, the burden of both stroke types has increased significantly between 1990 and 2010 in terms of an increased absolute number of people with incident stroke, number of deaths, and number of DALYs lost. Although the absolute number of incident ischaemic stroke was twice that of haemorrhagic stroke, the overall global burden of haemorrhagic stroke (deaths and DALYs) was higher. Whereas the main stroke pathological type in high-income countries was ischaemic stroke, most stroke burden worldwide was due to haemorrhagic stroke.

Second, the bulk of stroke burden in terms of incident events, deaths, and DALYs lost is borne by low-income to middle-income countries. These countries were particularly disproportionally affected by burden of haemorrhagic stroke compared with high-income countries. By contrast with high-income countries, where the overall incidence, mortality, DALYs, and mortality-to-incidence ratio of both ischaemic and haemorrhagic stroke have declined in the past two decades in both younger (<75 years) and older (≥75 years) age groups, in low-income to middle-income countries incidence of both stroke types increased significantly (especially in people aged 20–65 years). The average age at which people had ischaemic and haemorrhagic strokes was 3–5 years younger in low-income to middle-income countries than in high-income countries. Roughly a quarter of all events of ischaemic stroke and about half of all those of haemorrhagic stroke are happening in people younger than 65 years, with 73% and 83% of them, respectively, residing in low-income and middle-income countries. In 1990–2010, the incidence of both stroke types increased significantly in adults aged 20–64 years in low-income and middle-income countries. Our findings of a greater proportion of haemorrhagic stroke in low-income and middle-income countries compared with high-income countries, noticeable geographical variation in the incidence of major pathological types of stroke, and diverging trends in stroke incidence between low-income countries (increase in rates) and high-income countries (decrease in rates) are in line with the results of a systematic review of population-based studies of stroke incidence.^1^ However, unlike findings from that review,^1^ we also noted significant changes in incidence of haemorrhagic stroke in the past two decades.

Encouragingly, although we noted a trend towards an increase in the incidence of ischaemic stroke, a trend towards reduction in mortality rates for both ischaemic and haemorrhagic stroke, DALYs, and mortality-to-incidence ratios took place in low-income to middle-income countries. This finding might show advances in diagnosis of stroke type, and more targeted health care in some developing regions in low-income to middle-income countries in the past 2 decades, particularly because low-income to middle-income countries might be more heterogeneous than high-income countries.

The discrepancies between countries of different income levels are probably driven by the occurrence of the epidemiological transition.^13^ In the past few decades worldwide, life expectancy has increased, childhood mortality has reduced, and health status has improved overall in many regions.^11^ Globally, ageing populations are driving increases in the incidence of both ischaemic and haemorrhagic stroke. In low-income and middle-income countries, diseases related to infection and undernutrition have been replaced with more chronic diseases such as stroke and heart disease as the leading cause of disease burden, but unlike many low-income to middle-income countries, most high-income countries have implemented improved prevention strategies and better health care for these chronic disorders.^14^ Moreover, industrialisation and urbanisation have led to changes in the nutritional quality of foods, with high-fibre carbohydrates and fresh produce being replaced with more processed carbohydrates and high-fat diets.^15^ The resultant increase in the prevalence of diabetes, together with increases in smoking rates and increasingly sedentary lifestyles, have contributed to increased atherosclerotic disease.^14^ During different phases of epidemiological transition, increased incidence of haemorrhagic stroke is expected, particularly in low-income and middle-income countries, because hypertension is the dominant risk factor for this stroke type.

We noted substantial differences between countries in incidence, mortality, DALYs, and mortality-to-incidence ratio for both stroke types. The alarmingly high stroke burden in China, particularly for haemorrhagic stroke,^16^ might be attributable to the increased prevalence of risk factors for this stroke type—namely high blood pressure and smoking—and an ageing population.^17^ A review suggested that haemorrhagic stroke contributed to more stroke burden in China than it did in high-income countries, but wide regional differences were reported in the incidence and type of stroke.^18^ Hypertension, diabetes, dyslipidaemia, and smoking are modifiable risk factors that have increased in China. In India, smoking has been attributed as the cause of many deaths, particularly in men, with vascular causes being among the main contributors to death.^19^

Eastern European countries have undergone many socioeconomic changes in the past two decades. In particular, in Russia, alcohol was strongly associated with adult mortality.^20^ More than half of deaths in Russian men are attributable to cardiovascular disease, with hypertension, hypercholesterolaemia, tobacco use, inadequate diet, obesity, insufficient physical activity, and alcohol being among the prevalent risk factors for death.^21^

The decline in incidence, mortality, DALYs, and mortality-to-incidence ratios in high-income countries is likely to be because of improved prevention, and acute and chronic treatment of stroke. High-income regions such as western Europe, North America, Australia, and New Zealand have increased efforts to prevent and diagnose stroke, which might be shown by the delay of stroke incidence to older age groups.^14^ Mortality-to-incidence ratios for ischaemic stroke in people younger than 40 years were significantly higher in low-income to middle-income countries than in high-income countries. This finding might show an increased prevalence of risk factors such as alcohol use; tobacco smoking, including second-hand exposure; and high blood pressure in this age group, or it might be due to chance.^22^ By contrast, the increased overall global burden of stroke in low-income and middle-income countries could be attributable to reduced levels of awareness of risk factors, low levels of primary and follow-up health care, and a scarcity of basic drugs and equipment for the prevention and treatment of stroke.^23^

To progress our understanding of the burden of ischaemic and haemorrhagic stroke, and to better inform large initiatives in health funding, further study is needed that is specific to low-income to middle-income countries in terms of improved identification of what subgroups of the population are at greatest risk (eg, by age, sex, and ethnic origin) and could be targeted for preventive efforts. High-quality community-based epidemiological studies in low-income and middle-income countries with early neuroimaging investigations to identify stroke types are needed across WHO regions because heterogeneity is likely to exist across large regions such as central Asia and sub-Saharan Africa. A systematic review recommended the establishment of sustainable systems to obtain stroke data shared by other non-communicable diseases, and a wide application of feasible and practical surveillance techniques (eg, WHO STEPS) particularly in low-income countries.^24^ Population-wide preventive strategies should be given priority because even modest changes in prevalence of risk factors (eg, reduction of blood pressure, smoking cessation, and reduction of salt intake) could contribute substantially to the cumulative population risk reduction.^6,25^ Interventions to reduce the burden of chronic disease in low-income to middle-income countries should be cost effective and financially feasible.^26^ Because of the overlap of risk factors related to ischaemic stroke and ischaemic heart disease, preventive efforts focused on these factors (eg, raised blood pressure, cholesterol, diabetes, smoking) would be a cost-effective way to target prevention in a wide population. Raised blood pressure is the strongest risk factor for both ischaemic and haemorrhagic stroke, and on the basis of the high burden of both types, prevention programmes should focus on control of blood pressure, including both individual screening and treatment but also population-wide lowering of blood pressure.

Tobacco control (via increased taxes, reduced advertising, and banning of smoking in public places), strategies for salt reduction, and evidence-based multidrug strategies to treat those at high risk of cardiovascular disease, would be relevant for prevention of both haemorrhagic and ischaemic stroke. Several successful and cost-effective campaigns have already been identified and need to be adapted on a wider scale worldwide.^26^ Government initiatives to encourage and support healthy diets and increased physical activity are imperative in countries of all income types. Similarly, engaging of food manufacturers to achieve these goals by reduction of salt levels and fat content in processed foods would need to be implemented at government level.^27,28^

Haemorrhagic stroke was once a major cause of death in high-income nations, but its importance has receded in those regions over the 20th century.^29^ Our results suggest that, in the first decade of the 21st century, haemorrhagic stroke remains an important cause of death and disability worldwide. With the possible exception of raised blood pressure, other risk factors for haemorrhagic stroke have not been as well researched as have those for ischaemic stroke. Hence, studies to elucidate risk factors for haemorrhagic stroke are a high priority for future epidemiological research.

Our study had several limitations. Although we made every attempt to include as much data as possible from low-income and middle-income countries, such data is scarce, particularly from ideal population-based studies. Therefore, some studies from these countries were not of high methodological quality. Because we applied standard methodological criteria for selection of studies across the 20 year study period (1990–2010), we believe that the quality of data was consistent for studies selected for the analysis. Stroke diagnosis is unlikely to have changed between 1990 and 2010. Most diagnoses have been based on clinical (WHO) criteria and where imaging was available for verification of pathological types on CT images. However, changes might have taken place in the quality of routinely collected data over time, including diagnostic accuracy of stroke occurrence and its pathological classification, especially in low-income and middle-income countries. To assess potential effects of incomplete and less than optimum methodological quality data even in settings where no data are available for a country, cross-validation and out-of-sample predictive validity tests and simulation studies (sensitivity analyses) have been done. These tests have shown that the modelling strategies used in the study were robust.

Low-income and middle-income countries might have had lower rates of neuroimaging investigations than did high-income countries, thus reducing the ability to distinguish between types; as such, stroke incidence by type might be under-reported, particularly in rural areas of low-income and middle-income countries. For example, in the Trivandrum stroke registry study in India, brain imaging was unavailable for 44% of rural patients.^29^ Additionally, different modes of clinical presentation of ischaemic and haemorrhagic stroke could have affected the chances of investigators undertaking neuroimaging studies in low-income and middle-income countries towards higher rates in neuroimaging studies in most patients with severe disease, thus introducing a diagnostic bias. However, the effect of such bias is unlikely to be substantial because most estimates of the burden of ischaemic and haemorrhagic stroke were approximated from studies reporting neuroimaging verification of pathological types of stroke in roughly 70% of the patients. Although we cannot exclude the possibility of errors in calculations of stroke disability weights, the high correlations of weights across settings suggest that there is a broadly shared set of common values for health losses due to stroke, thus increasing our confidence in the reliability of calculations of disability weights. Our study’s greatest strengths are in the systematic attainment of a large and globally representative dataset and in the use of an innovative methodology that takes into account present evidence about stroke. Therefore, despite the limitations denoted above, our findings provide a unique global perspective on stroke burden by type, and could be used as a vital source of information for future planning of preventive strategies for stroke worldwide.

## Figures and Tables

**Table 1:** Age-standardised incidence and mortality per 100 000 person-years and DALYs lost per 100 000 people for ischaemic stroke by country in 1990 and 2010

	1990			2010		
	Incidence	Mortality	DALYs	Incidence	Mortality	DALYs
Afghanistan	123·46 (79·12–177·54)	114·82 (84·30–185·09)	2043·52 (1481·61–3088·72)	146·75 (99·28–209·31)	117·32 (91·85–175·11)	2032·11 (1576·60–2886·65)

Albania	207·05 (136·39–297·44)	24·79 (22·43–26·61)	403·25 (362·53–436·33)	206·14 (137·96–295·90)	24·82 (19·48–28·49)	376·11 (301·21–428·25)

Algeria	114·43 (75·46–167·67)	68·03 (59·24–77·42)	1032·50 (915·68–1149·90)	132·16 (87·87–192·14)	52·51 (46·53–58·45)	743·03 (662·55–810·74)

Andorra	124·35 (82·26–180·14)	38·91 (30·65–46·02)	515·51 (425·67–598·11)	102·37 (68·71–147·29)	25·04 (19·77–30·88)	343·12 (279·97–411·83)

Angola	130·83 (87·64–185·57)	56·02 (42·36–85·34)	914·66 (685·08–1272·09)	159·78 (104·45–234·14)	51·78 (37·27–73·88)	804·47 (578·70–1073·96)

Antigua and Barbuda	138·03 (92·44–201·40)	33·85 (26·04–37·43)	566·51 (438·90–623·25)	135·86 (91·19–200·43)	17·38 (14·81–23·58)	304·44 (261·61–396·51)

Argentina	145·02 (92·92–214·96)	56·11 (42·83–60·54)	768·46 (603·26–820·37)	105·54 (70·12–152·50)	27·48 (23·16–41·50)	366·44 (323·59–515·43)

Armenia	198·51 (130·43–285·69)	45·61 (39·76–51·77)	665·18 (581·27–745·76)	212·64 (141·77–308·13)	41·17 (32·80–47·26)	609·06 (485·33–695·67)

Australia	98·11 (80·00–119·09)	39·05 (35·42–43·22)	432·98 (395·62–473·61)	76·27 (62·86–92·39)	20·51 (17·40–24·72)	222·28 (191·99–263·93)

Austria	127·84 (88·97–188·45)	46·92 (39·48–50·68)	556·56 (464·01–604·11)	101·51 (68·46–149·81)	17·77 (14·89–27·25)	229·79 (192·04–314·66)

Azerbaijan	199·90 (131·14–286·56)	46·28 (40·57–52·12)	710·85 (624·67–792·38)	215·46 (139·56–314·49)	40·04 (30·67–45·94)	622·35 (485·48–701·48)

Bahrain	52·48 (36·96–71·48)	36·58 (32·67–40·64)	588·81 (533·69–655·82)	57·20 (39·68–78·35)	18·19 (15·73–20·43)	301·05 (260·81–340·42)

Bangladesh	98·43 (64·40–139·90)	30·37 (23·18–40·55)	451·42 (352·40–599·58)	114·65 (73·51–165·19)	26·92 (18·39–37·92)	375·05 (275·86–522·38)

Barbados	176·96 (135·21–227·65)	45·81 (37·62–49·51)	624·57 (519·75–679·14)	168·00 (130·76–216·70)	24·29 (19·79–38·63)	331·29 (275·94–484·71)

Belarus	374·97 (310·93–447·27)	97·28 (83·83–105·99)	1400·76 (1208·85–1505·36)	424·19 (353·20–508·41)	95·98 (69·90–107·44)	1471·88 (1119·52–1637·97)

Belgium	128·33 (85·51–184·04)	51·82 (46·21–56·30)	610·28 (552·93–664·24)	103·23 (69·19–150·43)	24·97 (21·28–32·44)	308·30 (266·78–387·85)

Belize	143·25 (93·58–208·51)	41·85 (36·46–46·11)	589·04 (506·76–641·33)	138·68 (94·30–196·30)	31·61 (28·11–36·51)	455·49 (408·54–520·81)

Benin	123·88 (82·13–182·51)	42·57 (34·20–54·29)	680·40 (548·33–832·43)	140·88 (92·91–205·70)	43·30 (33·57–54·63)	682·47 (530·40–831·35)

Bhutan	102·58 (67·34–150·06)	28·40 (20·75–41·66)	425·27 (320·00–604·94)	118·28 (79·26–169·06)	28·94 (20·74–40·05)	417·28 (311·29–574·18)

Bolivia	89·35 (59·14–131·06)	39·10 (32·32–50·36)	649·83 (549·94–797·19)	85·24 (54·38–125·79)	27·80 (22·77–39·91)	445·56 (378·57–572·91)

Bosnia and Herzegovina	219·50 (147·30–322·87)	109·95 (101·14–116·78)	1386·39 (1277·27–1469·83)	211·50 (138·89–310·29)	76·88 (62·43–88·99)	951·87 (772·34–1101·87)

Botswana	147·72 (97·34–219·56)	46·44 (35·31–63·10)	680·28 (533·73–900·18)	162·61 (105·89–236·68)	27·56 (16·27–37·23)	429·80 (279·92–551·18)

Brazil	189·69 (146·96–242·19)	61·26 (54·88–65·11)	909·78 (819·76–961·14)	178·73 (135·70–230·65)	40·90 (37·40–48·07)	570·49 (527·46–664·13)

Brunei	180·76 (119·21–260·86)	51·49 (33·37–62·02)	684·83 (468·34–809·48)	129·27 (84·07–188·85)	35·22 (24·83–43·17)	449·55 (342·72–535·42)

Bulgaria	234·42 (152·70–346·67)	123·77 (114·18–129·74)	1626·98 (1489·99–1703·95)	222·16 (147·64–320·65)	106·44 (90·94–114·27)	1380·28 (1173·14–1472·21)

Burkina Faso	117·01 (79·41–169·49)	23·71 (16·88–36·32)	417·76 (297·50–606·01)	143·78 (94·82–210·21)	46·23 (32·66–59·30)	747·73 (520·43–937·53)

Burundi	130·40 (87·07–194·12)	77·46 (40·12–127·01)	1207·49 (621·24–2071·37)	152·18 (101·34–220·44)	68·26 (41·91–115·58)	1051·11 (631·89–1877·00)

Cambodia	136·77 (90·04–201·44)	46·30 (35·26–63·65)	766·77 (603·75–1037·40)	159·38 (104·29–232·28)	50·27 (40·26–64·95)	800·41 (664·40–997·52)

Cameroon	127·52 (79·79–185·75)	44·72 (36·99–55·39)	722·89 (610·37–875·46)	147·32 (98·91–211·88)	41·05 (33·43–50·43)	653·51 (547·35–774·10)

Canada	133·05 (86·31–193·70)	32·98 (29·87–36·82)	404·86 (361·82–462·44)	108·17 (70·11–154·35)	18·91 (16·16–22·44)	287·30 (230·79–363·78)

Cape Verde	125·33 (81·77–185·50)	59·26 (49·66–69·54)	915·35 (779·53–1040·99)	141·31 (94·39–210·80)	48·23 (36·76–61·73)	706·46 (541·67–888·11)

Central African Republic	139·23 (89·09–201·88)	63·25 (46·45–87·89)	1059·39 (765·24–1445·41)	169·80 (111·15–247·45)	61·26 (43·01–88·81)	950·19 (664·29–1336·93)

Chad	124·95 (82·42–183·80)	38·25 (29·65–50·97)	644·51 (508·41–846·01)	143·13 (93·60–207·71)	38·36 (28·81–49·67)	638·96 (477·67–814·44)

Chile	109·41 (83·73–139·96)	51·25 (44·43–55·71)	657·01 (567·96–701·35)	77·75 (60·72–101·05)	23·34 (20·53–28·35)	301·59 (269·53–356·66)

China	226·45 (170·15–295·26)	56·33 (40·82–83·26)	767·53 (547·02–1169·10)	240·58 (178·54–310·63)	46·71 (36·74–61·60)	612·36 (478·07–835·78)

Colombia	97·43 (64·00–140·16)	37·70 (34·05–41·02)	532·03 (492·14–575·36)	97·39 (64·89–141·97)	28·59 (25·17–34·69)	364·58 (327·57–434·15)

Comoros	132·69 (89·63–191·24)	78·11 (60·70–95·77)	1209·70 (949·83–1484·21)	153·46 (99·95–223·69)	64·31 (51·10–79·74)	967·72 (770·27–1189·45)

Congo	137·39 (90·98–194·56)	70·00 (55·82–88·02)	1095·65 (880·79–1325·17)	169·89 (112·56–250·17)	65·04 (53·05–79·84)	1000·83 (821·25–1203·96)

Costa Rica	93·79 (62·82–137·13)	17·36 (15·00–18·73)	262·73 (226·04–291·15)	92·28 (62·43–135·74)	11·60 (10·33–14·33)	170·66 (146·88–207·30)

Côte d’Ivoire	129·60 (85·05–188·29)	47·55 (40·66–56·98)	781·17 (680·49–914·90)	150·37 (97·86–219·05)	50·99 (42·51–65·39)	847·53 (717·02–1052·67)

Croatia	223·75 (144·23–323·76)	87·71 (82·15–93·93)	1127·75 (1059·04–1200·81)	209·82 (136·39–306·18)	56·71 (51·53–62·78)	694·70 (635·59–750·92)

Cuba	148·43 (100·02–215·34)	48·99 (43·17–53·32)	683·65 (611·42–723·97)	144·44 (94·91–207·81)	43·31 (38·32–49·03)	540·19 (486·72–596·91)

Cyprus	127·46 (83·83–182·84)	37·19 (30·51–43·28)	505·08 (432·16–572·15)	103·41 (69·65–146·22)	21·89 (19·14–25·73)	311·11 (273·58–359·32)

Czech Republic	234·26 (158·40–342·94)	124·75 (115·89–133·89)	1597·87 (1482·53–1719·91)	215·06 (145·33–312·04)	63·53 (58·08–74·57)	760·29 (703·10–885·82)

Democratic Republic of Congo	127·96 (84·82–186·24)	49·03 (39·19–67·43)	759·21 (629·24–1020·30)	163·54 (104·73–245·10)	54·36 (42·41–78·56)	836·37 (650·05–1189·79)

Denmark	151·26 (130·58–174·11)	33·96 (30·61–37·26)	442·27 (402·14–492·68)	121·39 (104·35–140·84)	24·02 (20·77–27·46)	294·87 (259·02–332·85)

Djibouti	130·41 (85·37–186·49)	59·92 (41·60–82·13)	919·26 (652·52–1230·10)	149·03 (98·79–216·05)	48·12 (33·89–67·63)	720·40 (516·16–1005·00)

Dominica	139·87 (93·68–202·23)	38·05 (31·89–42·07)	581·40 (502·91–638·70)	131·44 (83·24–189·73)	24·97 (21·94–28·45)	402·76 (357·19–454·36)

Dominican Republic	144·34 (95·09–206·41)	37·98 (35·82–43·78)	628·79 (591·17–710·71)	146·01 (101·10–201·34)	41·73 (31·43–46·04)	606·73 (482·11–667·83)

Ecuador	85·88 (55·40–123·43)	17·06 (15·95–18·76)	320·20 (297·16–346·87)	81·63 (53·79–120·97)	13·03 (11·60–14·21)	225·49 (201·81–248·00)

Egypt	116·40 (77·98–166·92)	59·43 (54·08–73·95)	1047·88 (961·54–1290·59)	135·78 (90·84–197·25)	51·70 (44·36–55·35)	887·97 (759·17–946·28)

El Salvador	95·65 (64·10–137·29)	28·25 (17·84–31·85)	411·06 (263·71–461·03)	94·21 (61·70–137·29)	11·86 (10·09–17·44)	174·26 (148·26–239·97)

Equatorial Guinea	137·46 (90·44–200·69)	61·99 (46·58–97·20)	997·36 (743·04–1497·24)	165·16 (110·22–241·76)	42·75 (22·16–66·14)	652·07 (354·69–957·68)

Eritrea	132·17 (88·23–189·05)	65·66 (43·95–89·62)	1004·47 (686·96–1357·41)	153·18 (98·83–220·46)	57·70 (44·83–75·89)	861·80 (677·76–1125·46)

Estonia	453·72 (382·56–533·59)	123·71 (111·57–131·41)	1732·03 (1527·49–1838·03)	479·24 (403·87–556·40)	61·32 (54·59–77·28)	863·10 (771·71–1050·82)

Ethiopia	124·30 (82·03–179·55)	38·98 (27·54–55·52)	647·28 (472·33–894·86)	142·44 (94·84–209·85)	36·18 (28·57–46·58)	563·51 (451·74–723·45)

Federated States of Micronesia	162·47 (108·25–239·44)	83·11 (63·80–117·64)	1579·40 (1200·77–2093·35)	134·78 (88·09–196·26)	74·29 (54·22–104·30)	1343·94 (975·11–1804·38)

Fiji	171·55 (112·01–255·29)	138·55 (74·06–167·09)	2257·45 (1127·61–2712·22)	140·38 (91·86–207·65)	45·33 (35·88–80·33)	694·11 (569·83–1189·50)

Finland	215·99 (187·63–249·96)	50·33 (43·68–54·86)	646·94 (568·10–697·84)	173·86 (149·75–200·59)	24·22 (21·54–28·86)	315·65 (280·18–370·38)

France	103·84 (86·98–121·60)	24·93 (21·82–27·38)	333·00 (298·32–374·73)	83·56 (69·61–98·86)	12·98 (11·38–16·46)	204·23 (171·58–251·21)

Gabon	135·32 (89·01–201·29)	59·68 (42·05–76·30)	930·71 (678·56–1152·98)	168·21 (110·03–241·96)	55·00 (39·47–68·81)	827·07 (619·90–1016·05)

Georgia	135·30 (100·94–175·26)	34·13 (25·91–38·80)	652·91 (498·72–742·27)	141·90 (105·32–187·30)	24·80 (21·35–26·96)	503·14 (435·04–549·78)

Germany	176·31 (151·01–204·40)	48·11 (38·99–51·89)	573·82 (485·52–610·07)	141·66 (119·49–164·84)	21·11 (19·15–24·12)	281·30 (255·83–304·77)

Ghana	124·43 (84·02–179·86)	47·51 (38·77–58·96)	756·44 (646·32–908·03)	147·20 (97·92–215·04)	53·74 (45·06–63·86)	840·61 (731·78–968·97)

Greece	114·55 (93·64–139·06)	59·76 (54·00–63·89)	674·40 (609·85–722·78)	93·99 (77·18–113·77)	38·26 (34·57–45·32)	399·68 (363·64–471·30)

Grenada	144·38 (92·83–212·87)	71·64 (57·50–78·59)	1057·60 (838·63–1147·84)	141·40 (95·73–201·85)	49·41 (43·42–61·39)	705·15 (626·38–875·92)

Guatemala	92·95 (60·72–138·61)	25·70 (23·29–32·54)	374·61 (341·33–463·64)	94·50 (63·73–138·93)	19·27 (17·22–22·24)	307·77 (276·15–347·37)

Guinea	125·66 (81·75–184·21)	45·10 (36·22–59·27)	748·54 (612·65–951·02)	146·10 (96·62–211·84)	45·60 (36·08–59·82)	727·01 (589·53–918·22)

Guinea-Bissau	128·29 (83·85–182·22)	47·74 (36·36–64·36)	806·04 (622·04–1059·09)	146·79 (96·91–209·14)	49·03 (38·16–68·20)	802·14 (635·75–1098·98)

Guyana	149·64 (99·13–211·41)	97·05 (82·69–103·43)	1640·23 (1375·92–1745·31)	146·17 (96·57–210·23)	75·54 (66·72–89·28)	1174·35 (1035·09–1454·73)

Haiti	143·43 (91·57–204·66)	95·25 (85·72–105·96)	1608·38 (1456·51–1796·98)	139·99 (90·96–209·14)	79·66 (70·96–91·06)	1327·33 (1189·57–1512·95)

Honduras	99·98 (65·65–145·04)	47·08 (40·23–51·62)	686·84 (587·57–740·09)	103·76 (69·55–149·23)	45·15 (37·10–53·36)	613·21 (512·50–724·53)

Hungary	292·88 (242·05–350·84)	110·04 (98·81–116·19)	1612·57 (1425·66–1696·89)	271·23 (224·61–328·53)	60·02 (54·76–73·86)	843·17 (774·06–1038·42)

Iceland	129·88 (87·04–191·98)	28·59 (25·13–31·34)	379·72 (332·33–424·45)	101·52 (67·12–148·47)	15·48 (13·59–17·63)	218·98 (186·12–268·41)

India	128·48 (99·48–165·68)	37·44 (29·91–49·08)	571·39 (450·84–750·65)	143·45 (108·84–184·56)	38·83 (31·80–49·24)	556·56 (464·41–707·49)

Indonesia	129·71 (84·85–192·79)	71·91 (63·55–83·63)	1053·84 (938·47–1213·99)	149·53 (99·30–215·35)	77·39 (67·60–87·47)	1129·00 (1008·00–1271·85)

Iran	489·50 (394·07–606·17)	51·70 (43·69–61·62)	867·21 (741·41–1001·40)	570·82 (454·10–701·78)	39·79 (33·45–47·08)	589·58 (506·39–681·66)

Iraq	116·68 (78·23–170·03)	68·25 (57·60–77·60)	1085·84 (928·03–1213·34)	135·41 (89·10–199·70)	64·61 (56·24–73·02)	991·66 (870·06–1104·45)

Ireland	132·03 (89·44–194·38)	43·66 (38·46–47·40)	517·51 (460·52–565·73)	103·14 (66·89–150·89)	20·65 (18·28–26·12)	246·40 (214·55–300·53)

Israel	126·46 (84·31–183·75)	18·04 (15·82–19·26)	293·81 (260·06–330·10)	97·32 (65·25–137·66)	8·93 (7·94–11·08)	163·89 (132·32–207·45)

Italy	88·97 (77·70–100·81)	56·73 (49·75–60·68)	641·76 (566·86–679·84)	71·17 (62·12–81·51)	28·40 (24·70–36·21)	303·40 (274·13–382·48)

Jamaica	138·70 (90·04–202·21)	65·75 (60·93–70·60)	1093·59 (1014·53–1176·40)	138·14 (91·96–204·79)	52·53 (44·83–61·32)	807·27 (697·19–947·25)

Japan	176·15 (138·01–219·00)	42·74 (38·55–46·44)	525·86 (478·51–573·58)	128·65 (102·08–158·56)	24·63 (21·35–34·40)	311·20 (266·65–400·52)

Jordan	119·37 (78·88–174·83)	92·17 (82·20–103·57)	1371·33 (1234·35–1543·68)	137·40 (91·10–202·27)	62·86 (55·51–69·74)	881·51 (788·74–966·51)

Kazakhstan	199·93 (131·73–298·62)	85·20 (78·91–94·42)	1244·44 (1167·03–1373·69)	220·44 (144·78–318·03)	86·97 (73·13–97·59)	1339·38 (1102·21–1517·24)

Kenya	128·70 (84·94–188·61)	43·88 (34·02–56·36)	634·55 (509·80–806·04)	142·70 (96·76–206·87)	35·04 (27·46–44·46)	500·06 (403·63–632·34)

Kiribati	167·18 (104·89–251·55)	96·68 (84·04–106·88)	2001·91 (1788·88–2277·12)	132·18 (84·70–201·41)	81·41 (68·70–99·00)	1762·55 (1449·04–2143·13)

Kuwait	38·99 (27·99–53·84)	43·12 (39·31–51·44)	673·97 (623·75–774·00)	47·59 (33·03–66·96)	48·51 (34·44–54·83)	731·38 (523·39–815·60)

Kyrgyzstan	204·83 (129·55–305·74)	122·34 (109·82–131·83)	1759·28 (1575·60–1900·92)	227·71 (150·80–320·96)	103·98 (95·03–116·45)	1641·24 (1503·74–1819·56)

Laos	141·40 (92·87–204·98)	50·99 (38·12–70·32)	848·14 (647·68–1158·50)	164·38 (110·10–237·29)	52·66 (41·11–66·56)	846·51 (668·98–1058·72)

Latvia	337·72 (225·62–492·44)	144·03 (135·72–152·10)	1930·56 (1818·95–2039·24)	368·76 (245·77–535·20)	101·51 (94·39–109·95)	1361·22 (1254·15–1455·45)

Lebanon	113·03 (77·69–163·31)	44·69 (38·02–53·16)	755·41 (638·47–869·84)	131·63 (87·60–187·30)	29·28 (22·42–35·33)	470·63 (377·11–558·91)

Lesotho	150·26 (98·60–216·01)	59·36 (47·90–79·20)	929·42 (762·75–1204·89)	164·12 (107·17–233·76)	61·45 (46·77–80·83)	968·37 (743·00–1258·23)

Liberia	124·41 (81·18–182·37)	36·97 (31·35–46·15)	614·70 (535·12–734·60)	143·86 (95·30–210·85)	39·03 (31·69–55·15)	626·49 (527·11–843·73)

Libya	59·85 (45·91–78·39)	60·87 (51·19–71·94)	989·50 (852·64–1149·42)	70·05 (52·85–90·80)	56·45 (43·55–71·33)	847·68 (674·91–1060·66)

Lithuania	395·67 (336·83–465·73)	77·54 (71·64–87·33)	1111·25 (1029·92–1214·01)	433·97 (369·12–505·59)	65·69 (52·76–71·93)	929·62 (773·04–1021·53)

Luxembourg	122·27 (80·08–178·83)	34·85 (27·04–37·86)	466·85 (366·91–514·07)	97·37 (65·00–141·37)	12·19 (10·35–16·97)	185·50 (154·38–241·27)

Macedonia	227·71 (153·01–325·03)	139·43 (129·88–150·84)	1758·65 (1649·40–1889·33)	216·08 (146·03–317·97)	113·91 (100·93–123·90)	1406·02 (1258·42–1514·30)

Madagascar	139·89 (89·36–200·23)	95·49 (80·48–112·50)	1504·89 (1253·98–1797·84)	157·77 (105·31–233·09)	87·35 (67·96–107·84)	1338·27 (1034·18–1669·21)

Malawi	134·90 (89·57–192·72)	74·66 (50·39–101·01)	1173·60 (807·07–1544·80)	155·67 (102·02–224·98)	73·11 (50·44–97·22)	1128·13 (785·53–1499·41)

Malaysia	142·58 (93·63–205·06)	41·01 (36·88–46·39)	677·14 (617·90–752·95)	160·29 (103·87–228·78)	32·97 (29·11–36·66)	539·21 (488·92–587·44)

Maldives	137·82 (86·14–209·82)	49·79 (40·11–75·61)	882·17 (727·14–1290·92)	155·63 (99·97–228·17)	30·76 (22·27–35·88)	514·21 (391·33–605·07)

Mali	126·79 (83·89–189·18)	47·35 (38·01–64·75)	794·68 (645·37–1067·58)	144·73 (94·62–206·39)	45·58 (36·56–58·62)	737·44 (609·16–930·12)

Malta	129·14 (84·65–185·22)	39·29 (35·48–42·40)	541·84 (489·60–588·38)	105·33 (67·81–156·75)	26·29 (22·86–29·36)	327·67 (293·11–370·69)

Marshall Islands	164·79 (104·55–240·28)	75·21 (61·52–94·23)	1414·07 (1160·51–1757·14)	134·71 (89·59–188·98)	62·81 (50·85–79·54)	1212·80 (989·35–1515·72)

Mauritania	125·24 (84·42–181·90)	43·59 (34·23–56·76)	731·49 (583·50–937·25)	143·69 (94·71–204·47)	40·51 (31·33–51·69)	666·70 (520·22–831·75)

Mauritius	148·20 (98·10–214·48)	90·65 (83·47–97·73)	1363·82 (1241·30–1454·40)	163·84 (107·18–238·70)	49·38 (43·22–67·42)	639·49 (562·49–865·81)

Mexico	93·30 (60·91–138·79)	27·83 (25·01–30·57)	381·05 (348·32–415·81)	96·53 (63·37–140·78)	21·17 (18·91–23·63)	303·79 (272·18–338·00)

Moldova	334·09 (226·47–480·15)	99·00 (85·84–105·15)	1393·58 (1238·66–1480·19)	372·76 (245·03–539·36)	84·98 (75·44–90·81)	1309·11 (1143·44–1396·41)

Mongolia	195·75 (130·62–291·84)	10·58 (8·10–21·30)	260·70 (201·08–504·29)	215·68 (140·61–318·37)	12·05 (8·75–23·74)	290·91 (214·06–551·35)

Montenegro	208·52 (142·10–298·30)	25·02 (20·68–34·87)	399·34 (343·32–536·03)	202·23 (137·51–295·33)	32·04 (27·60–36·50)	476·15 (408·08–530·06)

Morocco	112·40 (74·11–166·19)	65·74 (57·70–76·15)	1003·72 (891·46–1160·66)	129·40 (84·35–182·98)	51·88 (45·36–59·45)	772·66 (690·20–868·30)

Mozambique	118·32 (76·82–171·82)	36·48 (27·09–50·06)	557·01 (416·57–767·58)	137·14 (89·97–204·35)	40·44 (29·84–50·79)	628·08 (459·78–787·23)

Myanmar	133·45 (88·36–194·21)	62·17 (45·49–89·62)	972·84 (708·89–1399·10)	151·86 (100·65–229·09)	60·42 (45·66–81·70)	926·06 (712·82–1268·84)

Namibia	155·54 (103·69–226·93)	64·25 (52·81–77·97)	1011·77 (834·91–1201·91)	167·56 (112·68–242·49)	59·46 (47·57–71·05)	923·70 (747·16–1085·04)

Nepal	102·97 (65·97–148·66)	29·56 (21·37–41·81)	450·08 (331·19–627·58)	114·24 (76·54–169·55)	32·42 (24·31–43·57)	469·04 (357·72–621·40)

Netherlands	105·14 (85·81–128·33)	26·36 (23·67–28·87)	357·03 (324·42–403·36)	83·51 (67·37–100·62)	14·70 (13·02–17·31)	221·16 (190·88–260·26)

New Zealand	101·69 (82·65–124·14)	33·69 (30·92–37·55)	401·55 (374·34–435·44)	79·45 (65·14–95·90)	18·03 (15·26–20·47)	211·54 (190·37–233·16)

Nicaragua	96·85 (64·71–138·64)	47·73 (35·89–52·11)	669·87 (507·03–728·71)	99·71 (65·79–142·11)	32·35 (28·42–39·91)	419·84 (375·98–521·79)

Niger	123·51 (82·89–179·36)	40·13 (30·96–54·16)	697·57 (536·42–894·12)	143·13 (92·13–206·42)	39·37 (24·47–57·14)	655·93 (414·11–938·39)

Nigeria	124·34 (84·76–178·73)	37·56 (31·63–47·09)	615·01 (525·20–724·97)	140·05 (93·96–209·06)	32·51 (24·16–41·84)	516·62 (394·63–636·64)

North Korea	163·69 (104·62–243·43)	51·56 (39·94–73·01)	708·04 (560·24–1029·28)	170·70 (112·65–250·63)	48·65 (35·67–80·09)	676·94 (492·18–1107·85)

Norway	135·58 (114·97–161·62)	31·33 (27·93–34·05)	400·85 (361·27–437·93)	108·48 (89·94–129·51)	17·23 (15·35–20·74)	225·64 (197·05–265·65)

Oman	113·15 (74·57–164·52)	60·65 (54·05–70·48)	962·67 (862·72–1092·41)	140·90 (94·83–197·85)	40·50 (35·20–45·27)	614·91 (529·34–680·07)

Pakistan	106·41 (69·75–152·70)	35·24 (27·63–47·00)	518·10 (410·11–677·60)	124·15 (83·25–179·80)	38·43 (30·07–51·63)	548·42 (441·38–723·96)

Palestine	199·12 (155·50–253·32)	47·94 (40·21–72·29)	807·65 (687·24–1143·78)	231·18 (180·64–289·83)	62·27 (48·89–69·23)	980·30 (754·12–1081·73)

Panama	98·63 (64·08–144·70)	46·28 (41·22–50·41)	602·28 (529·08–646·08)	95·52 (63·95–138·38)	33·64 (29·76–39·13)	423·28 (381·18–486·07)

Papua New Guinea	139·67 (89·70–202·18)	21·12 (12·03–55·30)	473·69 (273·31–1196·48)	121·57 (81·76–180·50)	20·63 (10·64–54·01)	456·31 (246·57–1121·38)

Paraguay	151·25 (101·55–214·61)	53·59 (44·76–57·86)	761·87 (643·01–815·00)	148·07 (98·49–216·43)	46·55 (41·35–56·96)	642·12 (578·57–780·23)

Peru	88·35 (56·04–133·26)	39·31 (28·61–42·95)	576·26 (452·63–620·74)	82·79 (55·45–118·27)	20·59 (17·74–28·80)	297·96 (267·06–374·22)

Philippines	136·85 (88·86–198·00)	34·32 (30·44–37·02)	584·44 (527·24–635·15)	162·29 (102·27–243·35)	46·72 (37·42–51·96)	767·79 (599·08–845·12)

Poland	186·61 (154·28–222·44)	78·40 (70·81–82·56)	1099·49 (985·17–1157·09)	173·18 (143·20–209·31)	51·06 (43·56–56·00)	682·83 (592·15–736·13)

Portugal	140·80 (116·84–171·18)	99·35 (84·28–104·63)	1150·99 (984·33–1210·02)	111·37 (92·24–133·22)	40·81 (34·32–61·48)	443·42 (379·75–637·78)

Qatar	46·52 (34·07–61·29)	31·27 (14·72–39·30)	481·27 (255·28–583·80)	51·88 (36·92–70·28)	9·17 (7·71–10·60)	178·34 (145·54–229·73)

Romania	224·83 (149·39–325·79)	116·29 (106·29–122·22)	1533·38 (1407·67–1607·59)	215·44 (145·30–313·28)	96·05 (86·82–102·67)	1253·57 (1114·25–1328·62)

Russia	332·19 (283·79–387·29)	155·05 (130·84–168·07)	2075·00 (1726·12–2251·33)	371·42 (316·04–430·26)	137·70 (108·71–150·90)	1909·12 (1509·93–2116·45)

Rwanda	132·24 (87·29–191·80)	81·01 (59·15–103·35)	1279·95 (937·76–1634·12)	148·10 (97·28–218·84)	53·87 (38·08–70·30)	821·87 (598·32–1075·62)

Saint Lucia	143·23 (95·16–205·59)	67·77 (58·62–72·79)	962·57 (842·06–1027·79)	138·77 (92·50–201·12)	49·99 (43·00–63·34)	590·41 (522·70–711·90)

Saint Vincent and the Grenadines	143·43 (96·22–215·22)	54·55 (46·89–59·43)	799·28 (700·95–864·14)	142·78 (94·87–207·97)	37·23 (33·36–42·87)	532·50 (483·42–605·56)

Samoa	154·97 (102·10–226·51)	72·00 (57·25–89·88)	1305·12 (1038·41–1580·51)	121·73 (82·03–176·12)	36·08 (26·00–47·32)	633·65 (485·53–806·62)

São Tomé and Príncipe	124·23 (79·90–185·26)	52·76 (45·57–61·11)	838·12 (749·29–948·56)	139·30 (92·94–202·58)	47·72 (34·67–58·61)	731·41 (534·64–880·55)

Saudi Arabia	75·98 (53·06–107·25)	58·82 (47·70–69·13)	893·63 (723·33–1028·57)	87·24 (60·18–118·66)	49·34 (39·57–56·03)	693·41 (550·38–775·91)

Senegal	120·62 (81·84–179·43)	27·07 (22·08–34·74)	502·52 (412·36–619·37)	136·78 (92·06–201·72)	23·10 (18·44–29·42)	406·29 (333·59–490·04)

Serbia	232·27 (189·36–279·75)	126·13 (110·44–139·97)	1673·92 (1468·73–1842·71)	223·15 (182·64–267·29)	100·02 (90·32–107·03)	1275·45 (1150·70–1356·54)

Seychelles	143·07 (93·87–207·08)	51·98 (41·18–58·69)	1014·60 (802·63–1154·87)	165·61 (107·93–241·63)	43·22 (29·29–51·85)	847·74 (604·91–1007·82)

Sierra Leone	130·10 (84·21–191·63)	48·72 (40·97–60·42)	861·85 (726·95–1036·72)	146·19 (96·30–216·95)	47·82 (36·64–62·68)	830·17 (628·20–1058·40)

Singapore	168·88 (112·44–243·12)	38·79 (34·58–42·05)	618·61 (556·59–661·72)	125·06 (84·32–180·23)	25·03 (20·81–29·86)	380·49 (334·51–436·60)

Slovakia	231·40 (152·01–339·38)	91·16 (84·54–100·41)	1264·42 (1181·78–1429·24)	216·15 (144·39–317·41)	62·38 (55·43–67·87)	846·43 (742·79–912·95)

Slovenia	227·00 (152·30–327·37)	103·66 (88·77–110·13)	1342·99 (1132·03–1425·23)	203·41 (135·47–291·41)	43·61 (39·64–49·65)	535·32 (491·65–605·20)

Solomon Islands	164·67 (107·42–247·03)	87·27 (68·51–127·21)	1657·95 (1324·68–2230·28)	137·24 (87·23–201·00)	83·64 (67·11–120·13)	1569·27 (1277·69–2034·37)

Somalia	131·44 (87·57–193·92)	63·89 (43·56–91·15)	998·64 (670·57–1469·73)	148·23 (100·02–212·47)	54·18 (38·44–75·79)	833·63 (587·30–1193·85)

South Africa	156·24 (100·83–232·34)	57·73 (46·82–63·82)	891·68 (727·57–980·54)	164·33 (108·79–239·90)	37·94 (31·86–43·98)	578·27 (509·44–678·09)

South Korea	170·86 (113·60–247·69)	135·92 (113·52–143·78)	1829·59 (1559·02–1924·56)	118·87 (77·06–172·16)	50·33 (43·84–68·38)	615·11 (546·79–806·32)

Spain	125·53 (82·75–178·37)	55·47 (45·96–59·68)	622·10 (534·40–659·20)	101·20 (68·00–145·08)	23·33 (19·80–34·49)	272·74 (241·86–368·36)

Sri Lanka	143·11 (93·26–211·56)	34·49 (30·34–37·30)	565·02 (502·87–615·42)	166·63 (105·68–242·79)	35·21 (27·90–41·53)	504·86 (410·32–593·02)

Sudan	127·58 (84·00–182·50)	46·66 (32·97–62·20)	681·34 (505·72–906·83)	141·87 (95·29–207·95)	28·29 (20·74–37·80)	408·53 (304·52–548·46)

Suriname	146·22 (98·00–213·25)	60·75 (56·03–69·25)	881·80 (819·21–1007·19)	134·21 (90·04–192·98)	54·76 (41·98–61·25)	766·81 (601·71–853·73)

Swaziland	150·58 (99·78–219·83)	56·66 (47·10–69·34)	885·79 (755·43–1058·65)	169·40 (111·58–251·25)	63·13 (51·63–75·70)	1016·68 (844·68–1185·45)

Sweden	152·49 (131·01–175·04)	38·13 (34·81–42·64)	465·41 (423·86–521·73)	123·05 (107·23–141·35)	24·07 (21·26–27·79)	277·38 (243·93–323·24)

Switzerland	125·86 (82·82–180·68)	34·72 (29·55–38·86)	415·95 (360·17–467·24)	102·40 (68·93–146·83)	18·22 (15·35–21·80)	221·75 (185·41–267·06)

Syria	116·40 (78·01–170·01)	33·97 (29·23–45·06)	597·94 (519·90–774·28)	133·55 (87·73–194·30)	25·92 (21·45–34·23)	438·68 (372·56–576·67)

Taiwan	175·30 (114·44–254·00)	46·11 (38·63–54·77)	631·55 (541·57–742·02)	171·33 (111·65–251·30)	25·50 (21·11–29·89)	365·87 (322·44–408·88)

Tajikistan	202·25 (132·22–301·74)	66·57 (58·54–74·87)	987·61 (872·24–1100·64)	222·52 (141·48–323·06)	70·44 (58·76–81·06)	1049·02 (889·42–1186·86)

Tanzania	145·35 (110·40–192·14)	32·94 (26·04–42·76)	492·57 (396·45–635·12)	164·72 (125·04–212·77)	27·31 (21·72–34·77)	401·02 (326·29–503·60)

Thailand	132·59 (86·81–192·40)	11·54 (10·23–16·66)	245·43 (219·41–313·90)	148·42 (94·76–214·80)	21·85 (12·88–25·45)	362·44 (247·55–408·31)

The Bahamas	141·24 (95·73–205·82)	48·54 (41·06–53·34)	705·01 (594·63–767·40)	123·26 (82·38–176·04)	17·31 (13·68–21·33)	298·03 (246·87–366·94)

The Gambia	129·99 (84·38–188·55)	54·55 (40·42–73·07)	939·71 (688·58–1238·98)	144·93 (96·57–211·43)	46·57 (34·56–60·37)	774·71 (569·25–992·07)

Timor-Leste	140·94 (94·35–207·72)	42·22 (35·70–52·93)	700·65 (596·06–861·25)	159·90 (105·03–230·49)	42·31 (35·50–53·99)	683·79 (581·08–835·97)

Togo	125·75 (80·72–186·47)	47·89 (40·34–58·83)	772·10 (667·60–921·22)	146·90 (96·07–214·15)	44·09 (36·44–57·15)	697·46 (586·36–859·85)

Tonga	154·47 (102·84–226·70)	43·79 (36·51–55·34)	736·97 (620·01–880·04)	125·87 (80·14–185·93)	29·00 (22·16–37·14)	496·39 (403·83–595·92)

Trinidad and Tobago	147·48 (97·88–211·86)	58·93 (52·82–63·31)	902·12 (818·24–961·59)	140·59 (93·82–204·10)	49·12 (43·56–56·99)	694·70 (621·57–796·41)

Tunisia	114·35 (75·12–166·26)	52·71 (44·03–68·66)	857·27 (727·22–1085·52)	131·74 (87·89–196·63)	42·22 (34·70–53·76)	643·58 (544·73–793·48)

Turkey	118·76 (77·04–172·36)	85·85 (74·86–98·55)	1584·20 (1381·62–1798·93)	134·52 (89·34–194·56)	52·58 (45·23–60·66)	876·02 (755·98–1015·71)

Turkmenistan	204·63 (134·72–293·46)	39·84 (33·18–53·11)	651·03 (552·85–875·89)	224·97 (147·90–331·72)	36·88 (30·56–48·08)	597·69 (504·09–783·60)

Uganda	126·96 (84·55–187·20)	48·78 (32·22–69·83)	729·36 (513·73–1039·60)	149·77 (99·00–216·38)	48·75 (33·16–65·56)	729·75 (510·40–979·97)

Ukraine	489·09 (397·46–593·28)	129·68 (106·36–139·89)	1754·54 (1467·52–1894·03)	533·40 (435·20–649·73)	98·48 (82·02–109·55)	1416·57 (1193·93–1564·73)

United Arab Emirates	117·83 (77·64–168·75)	64·65 (54·43–75·38)	909·18 (749·83–1046·47)	137·48 (88·73–208·76)	44·52 (34·11–54·82)	597·00 (453·16–720·42)

UK	107·73 (92·42–123·44)	45·64 (39·88–49·66)	533·70 (476·53–579·14)	85·22 (72·87–98·49)	24·15 (21·24–28·37)	276·52 (243·86–325·32)

USA	173·61 (135·82–216·70)	31·19 (28·23–34·75)	406·58 (378·82–446·68)	143·11 (112·54–177·75)	19·06 (16·84–22·43)	295·76 (270·88–325·55)

Uruguay	143·59 (93·67–205·84)	54·49 (50·14–60·54)	651·16 (608·20–714·45)	107·02 (69·94–157·65)	40·32 (35·13–46·66)	469·94 (419·19–521·25)

Uzbekistan	209·11 (136·77–303·30)	88·91 (79·87–99·84)	1471·01 (1326·31–1626·00)	232·09 (153·46–341·62)	90·59 (72·26–103·99)	1416·15 (1126·03–1591·91)

Vanuatu	165·23 (108·98–251·57)	91·57 (66·01–133·65)	1761·23 (1257·30–2505·95)	140·83 (95·71–207·38)	90·83 (63·54–136·11)	1668·40 (1173·50–2381·14)

Venezuela	96·97 (63·20–135·96)	34·71 (31·96–37·67)	514·70 (468·10–551·44)	98·91 (65·47–145·88)	24·89 (22·38–28·02)	348·51 (319·84–391·20)

Vietnam	117·64 (75·82–175·82)	11·56 (8·83–20·46)	226·49 (185·51–346·03)	135·07 (88·39–196·21)	9·72 (7·75–15·16)	191·27 (166·51–253·89)

Yemen	119·42 (80·21–176·17)	61·01 (45·34–93·61)	1100·37 (819·21–1575·56)	139·90 (94·80–198·95)	54·53 (42·72–76·11)	918·91 (741·67–1239·23)

Zambia	131·70 (86·55–194·13)	61·17 (45·98–84·84)	907·95 (700·54–1203·86)	153·96 (100·60–221·64)	64·91 (48·27–86·31)	968·73 (745·39–1235·93)

Zimbabwe	146·19 (110·75–187·21)	38·73 (32·14–47·24)	576·93 (495·19–688·81)	163·63 (125·69–206·52)	51·30 (38·04–72·22)	787·20 (598·89–1088·91)

Data are point estimates (95% CIs). DALYs=disability-adjusted life-years.

**Table 2:** Age-standardised incidence and mortality per 100 000 person-years, and DALYs lost per 100 000 people, for haemorrhagic stroke, by country in 1990 and 2010

	1990			2010		
	Incidence	Mortality	DALYs	Incidence	Mortality	DALYs
Afghanistan	32·38 (20·07–48·38)	140·36 (80·70–225·62)	3145·91 (1752·03–4983·92)	48·34 (30·99–75·08)	146·55 (87·89–218·10)	3194·66 (1866·82–4725·39)

Albania	87·83 (56·27–130·05)	119·31 (111·21–135·34)	1926·62 (1795·45–2146·28)	76·21 (47·24–114·70)	121·80 (104·38–137·36)	1777·90 (1532·66–2008·27)

Algeria	33·96 (21·18–51·61)	66·29 (57·34–74·64)	1384·16 (1173·98–1574·49)	48·14 (29·98–71·87)	47·04 (39·71–52·77)	884·13 (747·68–985·38)

Andorra	43·01 (27·89–64·51)	22·53 (18·62–27·44)	400·71 (335·67–488·56)	36·42 (23·09–52·77)	15·84 (12·80–19·57)	263·04 (215·98–319·57)

Angola	52·37 (33·71–79·46)	68·66 (47·87–100·54)	1626·45 (1087·86–2480·45)	67·91 (41·86–100·60)	55·22 (41·47–74·49)	1233·90 (897·03–1633·72)

Antigua and Barbuda	68·25 (42·91–100·86)	85·96 (63·49–98·99)	1572·05 (1146·98–1816·90)	63·79 (41·55–98·74)	43·76 (34·11–63·65)	743·96 (589·34–1067·01)

Argentina	66·74 (43·02–103·75)	39·89 (33·49–43·23)	1061·64 (880·40–1147·44)	50·72 (32·15–74·38)	24·73 (21·59–31·01)	592·24 (514·76–760·28)

Armenia	106·73 (68·91–158·01)	63·23 (55·65–70·11)	1110·03 (987·90–1227·58)	116·08 (74·97–169·26)	51·06 (42·88–60·15)	919·11 (765·09–1076·45)

Australia	31·50 (24·69–39·32)	18·01 (16·51–20·56)	364·87 (333·19–414·50)	25·09 (19·66–31·51)	9·34 (7·79–10·76)	175·94 (153·16–196·55)

Austria	42·80 (28·38–64·41)	31·76 (27·52–34·53)	571·64 (497·75–621·74)	35·70 (23·17–54·13)	15·63 (13·27–19·56)	262·27 (227·53–326·34)

Azerbaijan	107·86 (69·51–156·75)	81·51 (70·97–91·31)	1535·58 (1348·24–1737·51)	122·24 (77·40–184·87)	66·17 (50·86–77·54)	1207·62 (954·16–1397·14)

Bahrain	11·75 (7·86–16·76)	39·57 (34·96–45·30)	826·30 (719·36–943·44)	18·35 (12·19–26·93)	18·48 (15·95–20·99)	343·90 (297·80–385·64)

Bangladesh	56·95 (37·13–84·17)	37·84 (23·95–48·52)	731·58 (466·99–930·16)	53·60 (32·80–80·28)	27·87 (17·67–40·21)	506·02 (318·99–726·78)

Barbados	76·97 (56·33–102·55)	51·25 (42·36–56·44)	901·62 (729·89–998·39)	78·64 (57·95–102·74)	31·26 (24·97–43·17)	510·59 (423·39–664·13)

Belarus	50·91 (38·84–65·95)	38·64 (35·03–44·37)	943·22 (850·40–1038·97)	70·93 (54·42–90·44)	33·12 (29·41–37·44)	842·63 (735·32–942·29)

Belgium	40·92 (26·04–60·23)	17·01 (15·12–18·71)	399·86 (352·96–438·00)	35·73 (23·29–53·65)	11·72 (8·76–14·02)	248·32 (202·82–281·02)

Belize	57·25 (36·41–86·71)	48·19 (40·84–53·68)	944·01 (775·51–1048·84)	59·16 (39·05–88·18)	36·27 (31·90–42·01)	659·90 (587·09–770·98)

Benin	62·88 (41·19–92·74)	56·22 (46·46–69·16)	1339·64 (1035·03–1687·79)	71·66 (46·03–105·27)	52·37 (42·56–65·75)	1159·14 (929·98–1442·15)

Bhutan	46·60 (29·61–70·74)	34·27 (22·43–48·14)	692·47 (445·01–1001·88)	50·36 (31·64–73·49)	29·41 (21·02–42·05)	573·47 (403·05–827·87)

Bolivia	43·23 (28·46–64·37)	62·17 (51·81–75·36)	1416·23 (1153·63–1705·81)	37·97 (23·78–57·92)	40·29 (33·06–53·32)	864·46 (704·43–1110·46)

Bosnia and Herzegovina	78·98 (51·41–121·84)	95·10 (84·93–102·68)	1644·97 (1467·06–1790·23)	67·49 (43·57–99·37)	56·74 (45·85–70·11)	949·16 (746·78–1158·57)

Botswana	69·73 (45·81–106·21)	54·04 (41·37–71·73)	1078·87 (826·02–1425·30)	86·31 (55·25–125·77)	30·73 (20·14–45·15)	702·49 (501·15–1021·92)

Brazil	73·82 (55·22–98·40)	59·11 (51·33–63·82)	1387·87 (1196·75–1497·00)	65·23 (48·15–87·12)	38·01 (34·45–43·50)	795·16 (727·96–922·21)

Brunei	66·02 (42·63–97·83)	47·11 (36·47–66·29)	977·16 (775·16–1316·98)	47·98 (29·66–73·74)	33·41 (26·16–43·75)	653·60 (526·32–838·39)

Bulgaria	76·40 (48·40–117·11)	88·61 (78·12–95·27)	1769·53 (1545·61–1894·97)	64·62 (40·63–92·73)	56·87 (47·85–74·54)	1155·49 (986·22–1486·86)

Burkina Faso	62·49 (41·68–90·68)	35·24 (25·83–52·57)	836·07 (612·03–1206·25)	73·77 (47·36–108·31)	59·15 (46·22–76·79)	1254·96 (977·13–1624·95)

Burundi	58·88 (36·80–89·45)	119·29 (49·79–238·90)	2640·01 (1110·21–5335·50)	85·78 (56·17–128·12)	97·13 (48·09–190·72)	2102·41 (1026·78–4246·36)

Cambodia	67·56 (42·78–100·94)	103·89 (66·39–133·23)	2205·84 (1407·92–2862·63)	90·87 (58·35–134·37)	87·50 (67·13–108·70)	1827·47 (1413·63–2257·56)

Cameroon	62·35 (38·77–94·39)	56·08 (46·25–67·29)	1332·60 (1092·78–1607·11)	73·19 (48·57–109·56)	50·31 (41·05–63·46)	1140·87 (927·59–1420·10)

Canada	36·11 (22·99–53·87)	12·36 (11·34–14·43)	297·98 (271·22–338·55)	30·34 (19·17–45·92)	7·07 (5·87–8·08)	172·03 (146·93–198·39)

Cape Verde	65·98 (42·13–97·94)	69·12 (58·38–84·95)	1535·00 (1245·29–1877·36)	78·16 (50·83–118·24)	50·03 (40·25–67·58)	1046·83 (833·51–1377·99)

Central African Republic	53·29 (33·35–79·41)	84·79 (49·20–118·85)	2137·54 (1234·35–3007·33)	71·70 (46·54–105·33)	72·06 (39·58–109·11)	1582·98 (863·23–2429·22)

Chad	64·01 (41·02–95·31)	54·12 (41·65–71·21)	1306·41 (941·93–1732·94)	73·82 (47·80–109·23)	48·55 (35·68–63·94)	1118·45 (793·66–1496·38)

Chile	58·26 (42·38–76·24)	43·21 (38·58–48·77)	884·19 (787·41–996·20)	46·93 (35·24–61·38)	22·36 (19·41–26·57)	443·90 (385·72–519·42)

China	121·33 (88·26–158·31)	110·70 (79·06–139·48)	2127·05 (1557·05–2669·67)	159·81 (117·90–211·92)	80·20 (63·78–97·92)	1489·11 (1192·70–1781·08)

Colombia	39·27 (25·42–59·58)	36·04 (32·66–39·99)	869·43 (784·64–970·85)	37·56 (24·16–55·05)	23·76 (21·33–28·17)	481·91 (434·18–574·34)

Comoros	59·91 (37·61–91·16)	110·41 (75·89–155·66)	2427·98 (1651·64–3415·09)	88·40 (54·90–133·28)	86·33 (59·88–134·03)	1810·09 (1248·99–2818·61)

Congo	53·00 (33·09–77·79)	79·04 (63·51–98·57)	1797·82 (1439·61–2207·65)	71·26 (47·06–106·80)	74·12 (59·62–90·92)	1670·18 (1335·20–2080·75)

Costa Rica	39·17 (25·18–58·37)	32·66 (27·13–35·94)	533·69 (444·77–576·23)	37·47 (24·62–56·47)	20·72 (18·15–25·75)	315·30 (282·86–377·05)

Côte d’Ivoire	65·08 (42·17–96·41)	62·95 (53·09–76·31)	1527·57 (1284·24–1837·16)	77·17 (49·64–116·24)	66·99 (52·03–89·17)	1570·58 (1193·09–2057·32)

Croatia	73·09 (46·69–109·64)	66·78 (60·49–75·06)	1347·08 (1213·87–1513·46)	61·80 (39·21–92·64)	36·85 (32·41–41·55)	676·27 (603·83–766·63)

Cuba	49·32 (32·28–73·14)	23·57 (21·31–26·30)	670·95 (590·64–735·04)	52·72 (32·62–79·01)	19·37 (16·80–21·37)	447·54 (398·33–526·01)

Cyprus	46·23 (29·97–69·32)	35·77 (29·99–41·83)	691·65 (554·70–815·41)	37·48 (23·90–54·48)	20·58 (17·90–23·61)	348·31 (303·80–396·36)

Czech Republic	65·56 (42·93–97·95)	42·87 (35·49–46·86)	861·81 (702·82–944·06)	54·36 (34·75–80·88)	18·27 (16·05–21·98)	346·51 (306·25–421·05)

Democratic Republic of Congo	53·34 (34·56–79·41)	66·79 (43·64–91·65)	1519·59 (958·73–2090·78)	74·31 (46·83–113·89)	76·71 (45·17–108·68)	1705·80 (1010·86–2446·15)

Denmark	49·74 (39·46–60·83)	31·87 (28·50–36·29)	571·92 (518·13–645·86)	45·93 (36·67–56·85)	21·39 (18·17–24·64)	342·82 (292·11–384·48)

Djibouti	55·95 (34·91–83·62)	73·86 (52·58–104·04)	1616·92 (1139·98–2313·28)	79·63 (51·95–117·27)	58·54 (39·22–84·33)	1213·71 (810·59–1730·60)

Dominica	62·59 (40·62–95·64)	51·95 (45·28–57·38)	928·89 (805·98–1020·71)	65·79 (41·60–96·32)	36·72 (31·10–41·52)	638·53 (543·74–713·44)

Dominican Republic	61·04 (39·86–90·45)	66·64 (57·17–72·38)	1303·47 (1105·08–1409·70)	60·98 (40·42–88·28)	50·06 (43·99–55·91)	872·75 (771·52–996·06)

Ecuador	43·24 (27·43–65·11)	49·31 (40·25–53·71)	960·03 (807·88–1031·91)	37·62 (23·99–55·16)	29·12 (26·09–34·15)	546·17 (490·13–617·37)

Egypt	32·51 (21·44–48·85)	82·18 (68·53–111·24)	1750·39 (1399·34–2471·82)	44·51 (28·95–66·85)	75·63 (62·68–83·93)	1740·15 (1349·96–1996·75)

El Salvador	41·78 (26·94–59·72)	40·54 (29·52–46·45)	871·35 (608·72–1010·87)	35·66 (22·18–53·66)	22·58 (19·38–29·78)	415·02 (363·05–539·86)

Equatorial Guinea	54·30 (34·75–81·58)	83·36 (52·45–132·67)	1956·43 (1169·59–3383·61)	67·67 (44·23–101·98)	39·87 (24·61–61·43)	912·56 (574·09–1410·99)

Eritrea	57·31 (36·98–86·90)	99·37 (58·65–134·84)	2158·52 (1247·56–2934·37)	82·60 (53·01–121·45)	74·53 (52·09–96·67)	1553·71 (1091·58–2042·22)

Estonia	56·38 (44·51–70·87)	32·57 (27·37–36·29)	757·38 (647·31–839·42)	77·51 (59·75–96·98)	15·32 (13·64–18·89)	362·36 (320·27–431·90)

Ethiopia	58·71 (37·05–88·48)	63·81 (39·97–86·13)	1452·71 (905·66–1957·68)	83·14 (54·83–124·22)	50·22 (34·01–66·05)	1058·38 (719·14–1382·45)

Federated States of Micronesia	54·09 (34·29–84·81)	85·56 (61·12–125·35)	1827·12 (1242·79–2833·48)	42·47 (25·60–63·41)	73·33 (51·89–104·97)	1495·77 (998·90–2240·12)

Fiji	52·15 (31·91–79·48)	116·44 (73·27–154·01)	2498·42 (1495·41–3358·43)	35·78 (21·47–54·19)	70·70 (56·57–92·74)	1352·59 (1073·57–1785·89)

Finland	76·79 (62·23–92·47)	25·55 (22·56–27·96)	564·36 (494·81–620·66)	65·58 (53·51–80·84)	12·85 (11·37–14·65)	275·97 (246·55–317·77)

France	39·22 (30·52–48·70)	25·96 (22·76–28·86)	469·01 (414·78–522·83)	33·04 (26·22–40·57)	14·29 (12·30–17·67)	249·02 (220·56–293·35)

Gabon	51·60 (33·10–79·78)	59·28 (42·22–87·19)	1373·64 (971·49–1993·70)	68·11 (43·10–100·52)	54·04 (41·73–70·86)	1178·20 (900·06–1542·79)

Georgia	114·11 (84·08–150·57)	134·54 (122·00–155·11)	2329·98 (2098·73–2678·33)	127·67 (94·78–167·06)	120·91 (100·66–131·77)	2158·84 (1773·80–2372·75)

Germany	57·76 (47·17–69·60)	20·65 (18·07–22·45)	409·94 (345·09–442·33)	49·55 (40·43–60·33)	11·53 (8·94–13·03)	206·28 (162·16–228·29)

Ghana	63·24 (41·72–96·66)	62·51 (51·23–74·02)	1435·02 (1142·08–1726·77)	76·00 (48·23–111·67)	66·26 (53·05–79·84)	1455·56 (1160·67–1758·99)

Greece	42·18 (32·40–53·79)	60·39 (54·23–65·86)	885·15 (789·71–971·71)	34·45 (26·57–43·01)	39·66 (34·96–46·30)	539·20 (486·25–619·74)

Grenada	63·85 (40·80–95·87)	76·30 (59·18–85·52)	1598·01 (1218·03–1805·26)	64·22 (41·96–94·85)	51·99 (44·69–66·42)	1021·89 (891·92–1313·61)

Guatemala	40·70 (27·03–61·26)	29·10 (25·22–39·47)	625·22 (543·29–850·26)	39·26 (25·69–57·94)	22·93 (19·69–26·14)	509·09 (434·28–571·93)

Guinea	63·43 (40·73–93·23)	63·69 (49·97–76·72)	1564·02 (1147·28–1916·25)	74·43 (48·10–110·60)	58·43 (45·91–71·57)	1316·32 (1005·35–1606·85)

Guinea-Bissau	64·76 (40·99–95·12)	64·36 (44·47–88·92)	1589·56 (1033·82–2214·40)	75·10 (48·01–108·41)	65·20 (47·78–93·60)	1508·11 (1063·77–2226·72)

Guyana	69·55 (44·09–101·81)	140·42 (117·46–152·61)	3049·34 (2488·00–3335·79)	66·69 (41·71–99·39)	100·27 (86·62–126·77)	1992·14 (1689·83–2592·39)

Haiti	77·62 (50·32–114·72)	176·83 (156·03–220·78)	3734·29 (3288·33–4675·20)	81·30 (52·69–120·80)	151·08 (122·82–170·69)	3025·42 (2489·78–3428·36)

Honduras	41·49 (26·31–60·77)	51·16 (44·58–56·45)	1282·80 (1099·89–1453·30)	41·03 (27·08–59·70)	44·64 (37·74–52·01)	953·58 (802·70–1127·74)

Hungary	87·70 (67·03–113·73)	54·04 (47·86–58·88)	1119·71 (979·37–1234·18)	69·65 (53·41–89·30)	27·98 (24·33–35·43)	551·64 (487·44–696·04)

Iceland	41·03 (26·58–62·48)	25·08 (21·81–28·18)	387·31 (337·11–428·17)	35·12 (21·80–53·30)	12·64 (10·87–14·73)	175·52 (153·47–199·26)

India	49·92 (36·57–66·34)	47·41 (35·42–60·28)	1005·95 (757·57–1276·15)	55·10 (40·29–72·61)	43·55 (33·92–53·83)	863·78 (667·23–1071·38)

Indonesia	83·22 (53·66–126·22)	116·48 (100·25–137·03)	2264·89 (1938·33–2687·76)	110·12 (72·96–161·63)	115·94 (102·34–132·33)	2253·24 (1993·79–2588·68)

Iran	85·45 (62·40–111·58)	49·32 (39·80–62·22)	1094·55 (899·59–1362·04)	118·49 (87·54–156·12)	33·28 (27·21–42·52)	597·35 (497·01–751·15)

Iraq	29·81 (19·56–44·65)	58·03 (50·07–68·52)	1215·04 (1035·01–1462·66)	42·51 (27·73–63·66)	56·59 (47·64–65·30)	1118·20 (946·45–1310·65)

Ireland	40·09 (25·43–59·71)	26·11 (23·44–29·82)	524·58 (471·14–595·45)	34·23 (21·71–51·87)	13·91 (11·83–15·68)	262·00 (227·21–292·88)

Israel	44·15 (28·22–65·07)	40·49 (35·11–44·35)	641·33 (561·85–703·92)	38·17 (24·55–57·02)	19·75 (16·90–25·20)	284·96 (250·71–352·41)

Italy	29·68 (25·04–34·80)	20·40 (18·40–22·93)	456·32 (406·40–505·63)	25·11 (21·06–29·75)	11·97 (9·82–13·44)	233·26 (207·96–258·69)

Jamaica	69·01 (44·06–103·96)	65·79 (60·14–73·08)	1129·81 (1029·47–1244·08)	71·37 (44·77–107·35)	48·74 (41·11–57·98)	788·02 (668·48–933·15)

Japan	86·32 (66·43–111·61)	27·94 (25·55–31·98)	590·92 (539·81–672·58)	63·82 (49·73–80·86)	18·78 (16·84–21·58)	395·45 (353·24–448·71)

Jordan	28·59 (18·03–43·47)	45·27 (39·32–54·05)	923·63 (796·41–1122·27)	39·63 (24·78–60·53)	26·70 (23·52–30·11)	505·74 (445·91–573·74)

Kazakhstan	115·73 (72·91–177·52)	91·80 (84·11–103·07)	1739·40 (1604·38–1988·89)	121·82 (79·00–181·33)	86·24 (72·10–99·73)	1745·57 (1458·58–1982·28)

Kenya	57·57 (37·53–84·98)	57·92 (40·75–78·85)	1193·23 (826·37–1646·54)	79·14 (51·07–118·20)	41·41 (29·30–58·35)	825·39 (570·11–1181·62)

Kiribati	58·98 (35·66–93·15)	99·64 (83·95–113·92)	2172·20 (1835·42–2531·39)	46·87 (28·84–68·67)	86·71 (68·73–112·06)	1912·64 (1455·89–2480·24)

Kuwait	8·52 (5·54–12·18)	19·09 (16·05–21·66)	384·58 (332·06–427·59)	12·04 (7·53–17·68)	13·82 (11·77–15·92)	306·23 (246·04–344·25)

Kyrgyzstan	115·75 (71·97–172·05)	77·01 (69·46–88·20)	1701·78 (1554·85–1949·66)	125·66 (81·91–186·14)	79·79 (69·68–87·94)	1887·93 (1633·11–2082·01)

Laos	68·88 (43·99–102·67)	102·71 (64·91–143·62)	2231·75 (1370·35–3148·88)	94·10 (61·25–137·73)	88·64 (63·03–119·79)	1871·37 (1297·34–2553·71)

Latvia	46·50 (29·37–68·34)	36·85 (32·10–41·72)	824·35 (721·80–928·11)	63·89 (40·81–96·28)	19·84 (17·93–24·03)	459·94 (416·96–547·94)

Lebanon	25·44 (16·38–37·96)	39·51 (31·07–54·27)	908·04 (711·60–1189·35)	34·80 (22·56–52·19)	23·68 (16·20–32·01)	483·61 (334·29–641·00)

Lesotho	75·52 (48·73–110·15)	84·43 (60·12–106·01)	1763·94 (1260·06–2235·85)	95·47 (62·88–139·65)	82·78 (60·03–109·84)	1852·42 (1335·01–2529·38)

Liberia	63·57 (40·60–97·61)	49·17 (41·99–58·27)	1251·85 (1025·57–1525·77)	76·11 (48·66–114·08)	52·87 (38·88–69·45)	1191·25 (860·92–1541·76)

Libya	14·68 (10·50–20·06)	50·32 (40·30–73·04)	1080·74 (870·33–1549·52)	21·36 (15·06–28·74)	44·99 (37·35–60·29)	866·78 (733·84–1123·70)

Lithuania	47·95 (37·23–60·91)	22·09 (19·34–25·22)	566·61 (496·15–647·61)	69·91 (53·46–89·25)	14·42 (12·60–16·05)	378·74 (329·23–419·35)

Luxembourg	50·13 (32·09–75·27)	57·61 (48·48–62·95)	840·59 (721·56–917·38)	40·78 (26·10–59·83)	27·78 (24·42–33·83)	387·27 (345·03–445·48)

Macedonia	85·45 (55·59–125·94)	115·15 (104·24–133·48)	1949·23 (1785·49–2234·89)	74·62 (48·29–115·23)	89·37 (74·94–100·19)	1482·28 (1244·62–1640·77)

Madagascar	62·46 (39·65–91·91)	132·15 (109·49–153·91)	3027·30 (2514·12–3503·92)	85·81 (55·40–127·22)	108·73 (80·30–138·18)	2405·47 (1750·41–3096·58)

Malawi	59·06 (38·27–86·58)	109·88 (68·22–146·25)	2471·91 (1474·05–3320·76)	83·55 (55·38–124·27)	93·23 (55·79–126·92)	2004·28 (1180·99–2751·18)

Malaysia	64·52 (40·59–95·27)	69·90 (60·64–78·98)	1395·26 (1205·09–1565·67)	81·39 (50·97–120·14)	51·30 (45·21–57·61)	941·20 (830·42–1058·26)

Maldives	69·88 (43·00–106·10)	34·56 (20·52–54·54)	801·63 (495·42–1262·54)	83·62 (51·73–125·20)	19·69 (12·84–26·85)	383·11 (259·12–501·57)

Mali	64·01 (40·87–96·31)	70·90 (47·27–89·21)	1692·08 (1083·04–2161·41)	72·92 (46·78–106·44)	62·21 (47·66–76·67)	1419·09 (1040·14–1756·29)

Malta	41·44 (26·35–61·87)	27·86 (21·00–31·94)	476·19 (367·78–535·97)	35·46 (22·96–53·25)	16·85 (13·83–19·27)	254·55 (216·03–287·85)

Marshall Islands	53·31 (32·66–81·29)	72·49 (54·47–100·55)	1514·81 (1134·73–2144·79)	40·74 (24·98–60·98)	62·23 (46·73–81·93)	1351·12 (1018·72–1820·99)

Mauritania	62·75 (40·90–94·30)	60·86 (46·04–74·13)	1419·40 (1017·07–1731·73)	73·88 (48·22–108·35)	53·73 (45·12–66·51)	1206·70 (1002·47–1474·45)

Mauritius	62·63 (40·18–95·35)	88·22 (78·25–98·62)	1831·98 (1619·89–2043·45)	81·52 (52·11–122·45)	46·31 (39·81–62·10)	873·44 (756·25–1173·03)

Mexico	38·63 (24·60–57·71)	22·71 (20·41–26·07)	519·69 (469·00–597·43)	37·57 (24·23–56·12)	18·76 (15·61–20·84)	403·42 (344·61–447·65)

Moldova	48·61 (30·56–73·62)	61·54 (54·99–70·90)	1414·42 (1265·07–1636·56)	69·30 (44·05–102·24)	50·87 (46·55–58·52)	1168·68 (1065·34–1347·79)

Mongolia	134·27 (86·73–198·39)	195·71 (172·64–214·86)	3835·64 (3387·14–4220·57)	147·61 (92·17–222·58)	210·56 (178·44–234·44)	4118·90 (3456·44–4590·27)

Montenegro	86·29 (54·51–126·07)	121·73 (100·50–142·71)	2150·08 (1704·93–2594·54)	73·86 (45·81–109·54)	92·18 (81·68–108·28)	1405·71 (1270·00–1623·20)

Morocco	33·45 (21·20–50·82)	55·20 (47·11–66·88)	1284·88 (1032·83–1568·27)	47·00 (29·22–70·38)	40·20 (35·26–47·63)	879·61 (751·12–1038·37)

Mozambique	58·59 (37·54–87·82)	47·08 (32·03–63·07)	957·46 (647·43–1293·10)	82·45 (52·96–123·34)	46·72 (34·91–61·16)	973·83 (728·18–1284·54)

Myanmar	82·44 (53·76–119·91)	129·23 (66·65–186·29)	2550·91 (1298·36–3780·29)	107·06 (67·85–165·67)	104·96 (60·01–152·44)	2045·19 (1134·39–3011·95)

Namibia	74·59 (47·92–111·16)	85·34 (69·45–103·16)	1810·14 (1461·74–2220·19)	91·30 (59·55–134·68)	70·51 (57·08–84·22)	1474·78 (1196·76–1758·93)

Nepal	49·25 (31·52–72·96)	41·47 (27·35–55·33)	852·99 (565·75–1132·83)	53·38 (34·43–81·17)	41·07 (28·64–54·84)	814·93 (560·17–1093·69)

Netherlands	35·89 (27·90–46·36)	31·31 (27·78–35·18)	506·34 (452·67–567·91)	32·29 (25·28–41·34)	17·83 (15·72–20·89)	274·08 (246·08–312·01)

New Zealand	32·98 (25·97–42·02)	27·94 (25·26–31·36)	512·85 (460·64–567·45)	26·09 (20·42–33·48)	13·64 (11·19–15·73)	228·84 (194·28–254·75)

Nicaragua	43·66 (27·60–64·14)	46·50 (38·84–50·51)	958·43 (778·30–1050·75)	40·88 (26·32–62·23)	37·15 (32·83–43·33)	680·60 (613·19–802·70)

Niger	63·44 (41·03–92·75)	61·54 (48·22–82·19)	1557·09 (1193·48–2057·92)	75·99 (48·09–112·22)	53·71 (39·11–75·71)	1234·63 (882·84–1755·42)

Nigeria	63·00 (40·33–91·61)	46·93 (39·63–57·50)	1139·24 (930·01–1370·28)	71·80 (47·75–105·59)	34·96 (27·73–46·07)	787·10 (622·03–1023·60)

North Korea	76·81 (47·70–115·69)	96·58 (66·93–129·46)	1932·22 (1347·69–2545·62)	113·35 (73·34–164·98)	100·95 (67·84–134·55)	2021·85 (1357·81–2681·88)

Norway	45·24 (35·85–56·11)	35·25 (30·67–38·94)	544·24 (481·33–603·55)	39·93 (31·85–49·21)	18·82 (16·36–22·56)	264·92 (237·92–307·38)

Oman	29·95 (19·21–45·29)	53·52 (42·19–78·19)	1127·60 (880·88–1689·35)	43·96 (28·37–65·65)	34·57 (28·68–46·47)	647·26 (543·84–851·95)

Pakistan	46·86 (30·33–69·30)	43·26 (31·10–54·16)	902·07 (637·46–1122·29)	53·05 (34·20–78·66)	44·83 (34·71–56·44)	918·76 (700·39–1159·68)

Palestine	70·68 (50·87–93·93)	160·10 (123·96–183·56)	2695·26 (2085·44–3170·88)	98·75 (73·64–129·98)	111·36 (90·76–122·93)	1812·97 (1476·46–2008·30)

Panama	42·60 (27·53–61·27)	41·80 (36·00–46·52)	794·43 (666·77–875·29)	42·10 (26·46–62·06)	31·80 (27·50–37·60)	556·56 (485·79–674·16)

Papua New Guinea	44·73 (28·18–66·88)	38·90 (17·55–89·55)	987·56 (404·90–2349·07)	37·30 (23·35–56·13)	35·41 (14·24–82·81)	896·80 (338·67–2186·15)

Paraguay	66·87 (43·92–99·72)	67·84 (58·75–73·41)	1283·31 (1111·28–1382·38)	60·31 (38·27–88·81)	59·88 (52·84–71·19)	1141·72 (1006·28–1348·51)

Peru	38·95 (24·73–58·02)	25·05 (21·54–27·76)	652·50 (565·88–722·09)	34·14 (21·90–51·03)	18·10 (15·00–20·73)	397·11 (317·25–457·52)

Philippines	63·46 (40·04–96·81)	55·37 (48·57–60·31)	1298·15 (1158·07–1410·20)	86·11 (54·44–128·07)	62·84 (52·23–69·71)	1404·09 (1154·76–1552·66)

Poland	67·17 (51·85–85·23)	103·91 (84·95–112·96)	1656·19 (1397·37–1781·00)	53·23 (40·57–67·33)	42·40 (34·70–62·24)	730·66 (636·92–965·39)

Portugal	63·73 (49·93–79·93)	70·99 (58·64–76·93)	1272·09 (1062·84–1372·14)	51·08 (39·53–63·76)	31·95 (25·96–43·16)	531·49 (446·27–690·67)

Qatar	11·07 (7·60–15·52)	35·30 (23·01–44·89)	642·27 (426·01–816·95)	14·55 (9·61–21·38)	13·21 (11·39–15·94)	228·07 (193·91–266·29)

Romania	76·44 (48·67–115·87)	84·47 (76·68–92·70)	1630·50 (1467·94–1776·19)	66·03 (43·68–98·07)	62·91 (57·04–72·90)	1158·05 (1057·38–1352·39)

Russia	44·22 (34·99–55·22)	48·85 (42·45–60·64)	1077·46 (947·62–1257·88)	61·98 (49·22–77·13)	42·07 (36·16–51·95)	984·31 (846·51–1153·35)

Rwanda	58·77 (37·85–87·88)	115·98 (72·06–174·63)	2619·76 (1625·12–3977·20)	82·94 (54·59–121·29)	65·93 (45·18–97·79)	1390·55 (940·59–2073·23)

Saint Lucia	66·78 (42·99–99·23)	75·86 (62·81–82·76)	1493·03 (1224·06–1631·94)	69·61 (45·11–105·35)	49·12 (42·26–61·48)	828·14 (719·22–1076·13)

Saint Vincent and the Grenadines	64·80 (41·78–98·88)	74·81 (61·63–82·82)	1350·31 (1117·30–1485·12)	63·69 (40·81–94·22)	48·30 (42·39–58·33)	828·83 (733·04–994·07)

Samoa	54·89 (34·50–81·20)	78·91 (54·29–138·00)	1634·85 (1098·33–2976·81)	42·89 (26·49–64·79)	38·24 (25·43–64·37)	750·84 (501·42–1279·31)

São Tomé and Príncipe	66·63 (42·02–99·60)	64·44 (56·95–74·16)	1603·26 (1374·12–1878·56)	77·79 (51·22–112·51)	51·19 (41·54–64·36)	1149·64 (931·41–1426·71)

Saudi Arabia	19·77 (13·07–28·92)	48·14 (40·75–57·05)	914·11 (765·69–1102·68)	28·54 (19·24–40·97)	39·60 (32·78–45·14)	693·54 (579·12–783·90)

Senegal	62·37 (40·43–94·15)	38·84 (27·67–48·73)	995·45 (677·52–1237·63)	71·28 (46·17–109·08)	29·28 (20·07–39·23)	697·77 (459·22–909·72)

Serbia	74·94 (58·88–95·55)	46·07 (39·14–54·10)	1043·61 (881·72–1233·81)	63·36 (48·55–81·04)	29·91 (27·17–35·02)	630·58 (570·13–751·23)

Seychelles	69·12 (43·95–103·50)	97·49 (85·62–116·66)	1960·06 (1715·79–2341·14)	84·11 (54·87–124·58)	64·67 (54·06–79·60)	1291·34 (1096·74–1556·40)

Sierra Leone	66·35 (42·88–98·22)	68·60 (56·14–86·92)	1764·68 (1441·59–2160·31)	77·18 (48·52–116·51)	63·25 (49·64–83·02)	1493·49 (1176·24–1928·42)

Singapore	60·17 (37·41–88·38)	43·60 (38·30–47·60)	888·80 (777·61–963·48)	44·19 (27·09–66·03)	22·88 (19·50–26·48)	423·67 (369·99–484·55)

Slovakia	60·26 (37·80–90·12)	37·59 (31·23–42·14)	815·83 (680·19–916·90)	48·61 (30·16–72·59)	18·45 (16·57–21·32)	394·83 (349·14–452·98)

Slovenia	62·70 (41·20–94·44)	20·08 (18·26–23·80)	529·13 (478·98–609·60)	54·37 (34·38–81·95)	10·64 (8·18–12·05)	239·31 (200·77–267·03)

Solomon Islands	56·89 (34·87–90·41)	103·78 (72·78–166·92)	2203·88 (1467·18–3745·73)	45·66 (28·35–69·58)	103·92 (72·78–183·19)	2212·52 (1474·80–4107·02)

Somalia	58·84 (37·68–89·02)	91·07 (54·06–138·65)	2001·11 (1185·95–3169·81)	83·58 (56·36–125·63)	73·62 (44·00–109·01)	1556·55 (897·72–2384·59)

South Africa	68·11 (43·62–103·37)	74·30 (56·10–84·77)	1599·62 (1168·81–1835·14)	87·41 (56·38–129·75)	46·01 (39·32–58·26)	992·31 (855·75–1314·38)

South Korea	76·67 (48·49–113·79)	82·17 (69·41–88·35)	1771·30 (1480·71–1903·95)	56·40 (35·64–82·98)	27·08 (23·03–34·40)	502·64 (438·80–636·30)

Spain	44·62 (28·97–64·48)	19·37 (17·48–22·07)	461·74 (406·85–518·50)	37·89 (24·83–56·11)	11·48 (9·49–13·04)	232·61 (205·26–261·44)

Sri Lanka	58·71 (35·56–88·55)	37·75 (33·27–42·48)	862·44 (756·44–957·19)	76·41 (46·98–115·86)	30·14 (24·71–35·76)	568·76 (471·43–668·07)

Sudan	55·18 (36·07–81·15)	59·15 (41·39–77·83)	1211·16 (816·91–1604·45)	78·15 (50·87–117·40)	31·56 (24·40–40·89)	606·22 (463·24–792·24)

Suriname	60·97 (38·78–90·52)	72·48 (66·11–84·60)	1402·32 (1282·78–1609·71)	68·64 (46·03–102·07)	59·50 (42·19–69·79)	1087·32 (782·07–1264·49)

Swaziland	70·63 (45·06–107·53)	71·67 (55·92–86·85)	1496·79 (1164·47–1828·40)	90·04 (57·42–134·81)	76·70 (60·46–91·43)	1711·61 (1363·69–2046·68)

Sweden	45·61 (36·43–55·96)	19·13 (17·40–22·20)	373·19 (340·46–429·95)	40·28 (32·21–49·20)	11·60 (10·19–13·09)	206·75 (182·66–234·94)

Switzerland	42·36 (26·93–62·95)	21·06 (18·19–24·25)	364·76 (321·14–409·43)	35·13 (22·78–52·29)	11·60 (9·69–13·88)	178·20 (151·74–207·62)

Syria	28·12 (18·21–42·19)	81·09 (69·28–101·75)	1783·33 (1531·60–2212·06)	39·90 (25·60–59·26)	57·14 (43·48–73·27)	1178·71 (913·17–1477·89)

Taiwan	75·67 (48·17–113·82)	61·87 (52·12–75·17)	1241·32 (1059·43–1501·85)	103·26 (64·43–154·74)	31·31 (27·17–36·14)	626·24 (539·08–715·26)

Tajikistan	118·08 (74·67–173·75)	100·58 (82·63–111·90)	1854·19 (1544·07–2037·64)	130·74 (83·13–197·37)	99·98 (75·54–115·10)	1834·22 (1400·71–2135·69)

Tanzania	63·46 (46·11–85·90)	42·12 (30·42–54·08)	842·81 (613·47–1072·85)	88·65 (66·29–118·53)	30·47 (22·09–40·39)	602·19 (434·65–799·86)

Thailand	60·82 (38·30–90·11)	50·98 (46·36–59·20)	1042·43 (934·30–1190·47)	86·71 (54·53–131·91)	40·98 (34·58–47·62)	745·67 (617·30–842·08)

The Bahamas	60·08 (39·21–88·72)	54·75 (48·04–60·85)	1092·09 (950·91–1202·25)	57·83 (36·99–86·04)	20·24 (16·41–24·65)	386·72 (329·53–461·38)

The Gambia	66·12 (41·96–98·24)	72·87 (48·77–103·28)	1784·42 (1140·76–2556·43)	74·74 (48·13–112·12)	56·22 (38·19–77·12)	1272·12 (842·34–1754·94)

Timor-Leste	68·09 (43·25–102·83)	80·84 (63·55–101·98)	1714·79 (1345·79–2161·32)	90·77 (58·53–135·33)	74·97 (61·43–95·66)	1552·52 (1280·45–1961·11)

Togo	63·59 (39·36–94·99)	64·43 (54·37–76·22)	1507·89 (1268·35–1781·72)	76·09 (48·89–112·89)	56·93 (45·74–71·50)	1269·49 (1023·12–1585·01)

Tonga	43·96 (26·64–67·27)	40·17 (31·34–53·68)	736·58 (559·75–1023·03)	34·73 (21·23–54·04)	25·35 (19·32–34·02)	472·33 (359·43–645·30)

Trinidad and Tobago	58·57 (37·52–85·29)	66·29 (58·06–72·33)	1381·74 (1201·77–1500·52)	58·30 (37·22–88·57)	47·42 (40·70–60·26)	885·18 (764·82–1115·63)

Tunisia	29·26 (18·80–44·52)	49·24 (35·97–66·71)	1037·10 (749·67–1394·29)	41·23 (26·52–61·62)	36·46 (27·21–49·91)	684·66 (510·75–918·06)

Turkey	31·88 (19·98–48·12)	86·59 (72·16–105·73)	2232·46 (1883·47–2676·85)	43·11 (27·71–64·35)	48·84 (41·02–57·91)	1115·81 (909·72–1325·63)

Turkmenistan	113·39 (72·18–165·80)	118·90 (104·96–127·56)	2501·36 (2218·82–2663·97)	122·50 (78·73–184·04)	101·60 (84·50–117·40)	2081·03 (1717·38–2419·32)

Uganda	57·23 (36·50–85·86)	59·23 (33·75–87·68)	1291·39 (729·16–1902·39)	81·25 (52·94–120·81)	49·36 (31·29–71·54)	1022·84 (636·60–1484·18)

Ukraine	68·19 (51·08–88·54)	42·44 (38·74–48·70)	960·88 (871·95–1065·18)	88·61 (68·21–114·21)	31·92 (28·20–37·06)	752·98 (656·08–842·01)

United Arab Emirates	27·41 (17·57–40·84)	47·38 (35·97–71·18)	880·78 (658·87–1320·96)	39·90 (23·69–62·76)	33·00 (24·29–46·22)	556·49 (398·72–777·45)

UK	34·24 (28·26–41·50)	25·56 (23·15–28·59)	519·62 (468·66–579·08)	30·18 (24·70–36·60)	14·07 (11·81–15·71)	265·46 (233·03–298·43)

USA	47·05 (35·19–61·19)	14·17 (13·05–16·49)	363·74 (333·94–418·12)	41·50 (31·27–54·01)	9·64 (8·02–10·88)	244·64 (211·74–270·17)

Uruguay	69·68 (44·89–104·75)	49·18 (44·39–54·29)	1091·88 (981·97–1203·16)	56·67 (36·13–83·98)	32·70 (29·01–37·56)	700·65 (621·56–794·78)

Uzbekistan	107·56 (69·37–162·76)	71·39 (64·55–82·60)	1477·76 (1346·84–1691·67)	118·68 (76·53–177·70)	67·45 (58·42–78·94)	1315·77 (1142·14–1557·77)

Vanuatu	55·52 (35·41–86·38)	95·15 (66·56–144·08)	2036·96 (1356·54–3292·51)	45·07 (28·63–67·23)	92·76 (64·18–138·74)	1927·40 (1285·56–3048·34)

Venezuela	39·72 (25·44–56·81)	43·00 (37·78–47·38)	868·61 (761·27–956·67)	38·64 (24·51–56·73)	29·72 (26·23–34·31)	576·03 (518·11–671·29)

Vietnam	95·94 (62·08–142·34)	159·56 (121·07–182·13)	2572·06 (1935·45–2942·07)	119·71 (78·89–177·22)	114·77 (89·56–131·54)	1763·67 (1386·67–2029·02)

Yemen	31·73 (20·28–47·90)	72·28 (44·87–115·97)	1736·69 (1089·34–2663·97)	46·31 (30·01–68·16)	62·70 (43·53–93·46)	1361·41 (949·48–1966·49)

Zambia	56·44 (35·51–85·74)	71·19 (52·05–91·20)	1440·84 (1038·28–1851·49)	81·86 (51·66–118·80)	69·38 (53·14–90·51)	1391·56 (1038·13–1831·28)

Zimbabwe	67·30 (48·84–88·82)	45·15 (37·93–53·82)	946·51 (800·48–1135·37)	89·14 (66·26–115·40)	64·52 (47·90–87·33)	1434·77 (1051·30–1978·22)

Data are point estimates (95% CIs). DALYs=disability-adjusted life-years.

**Table 3:** Age-adjusted annual incidence and mortality rates (per 100 000 person-years), mortality-to-incidence ratio (MIR), and DALYs lost for ischaemic and haemorrhagic stroke, by age group in high-income, low-income, and middle-income countries, and globally in 1990, 2005, and 2010

	1990		2005		2010		p value*
	n	Point estimate (95% CI)	n	Point estimate (95% CI)	n	Point estimate (95% CI)	
**High-income countries**							

Aged <20 years							
Ischaemic							
Incidence	8268	2·46 (2·25–2·67)	6680	2·21 (2·00–2·43)	6110	2·11 (1·91–2·33)	0·013
MIR	..	0·035 (0·031–0·041)	..	0·031 (0·022–0·037)	..	0·025 (0·017–0·031)	0·006
DALYs	35 027	10·42 (9·49–11·77)	28 444	9·42 (6·92–10·83)	22 912	7·89 (5·68–9·66)	0·008
Mortality	384	0·11 (0·10–0·13)	291	0·10 (0·06–0·11)	223	0·08 (0·05–0·10)	0·003
Haemorrhagic							
Incidence	5107	1·52 (1·40–1·65)	3735	1·24 (1·13–1·36)	3393	1·17 (1·07–1·28)	<0·001
MIR	..	0·289 (0·238–0·335)	..	0·196 (0·160–0·232)	..	0·163 (0·127–0·198)	<0·001
DALYs	154 507	45·98 (37·73–51·49)	71 713	23·76 (20·49–26·98)	55 326	19·06 (15·66–22·17)	<0·001
Mortality	1974	0·59 (0·48–0·66)	907	0·30 (0·26–0·34)	691	0·24 (0·19–0·28)	<0·001
Aged ≥20–64 years							
Ischaemic							
Incidence	619 121	87·16 (81·02–93·66)	663 271	85·60 (79·89–91·93)	743 213	93·82 (87·23–100·81)	0·060
MIR	..	0·142 (0·122–0·157)	..	0·114 (0·098–0·128)	..	0·092 (0·077–0·104)	<0·001
DALYs	2 818 833	396·84 (353·88–423·46)	2 650 292	342·02 (302·66–371·93)	2 451 018	309·40 (272·20–340·16)	<0·001
Mortality	87 833	12·37 (10·74–13·29)	75 345	9·72 (8·37–10·66)	68 140	8·60 (7·31–9·59)	<0·001
Haemorrhagic							
Incidence	343 294	48·33 (44·87–52·17)	379 159	48·93 (45·35–52·75)	409 193	51·65 (47·80–55·32)	0·105
MIR	..	0·465 (0·407–0·519)	..	0·368 (0·320–0·413)	..	0·294 (0·255–0·335)	<0·001
DALYs	5 369 589	755·94 (673·55–821·03)	4 878 882	629·62 (560·28–688·56)	4 148 598	523·70 (463·91–581·56)	<0·001
Mortality	159 383	22·44 (19·89–24·48)	139 460	18·00 (15·97–19·81)	119 958	15·14 (13·30–16·94)	<0·001
Aged 65–74 years							
Ischaemic							
Incidence	1 013 498	1212·57 (1122·02–1302·50)	1 207 812	1136·70 (1059·80–1220·27)	1 206 882	1104·11 (1027·09–1183·37)	0·031
MIR	..	0·188 (0·159–0·206)	..	0·177 (0·154–0·196)	..	0·143 (0·126–0·158)	<0·001
DALYs	3 781 762	4524·60 (3941·34–4749·17)	4 348 756	4092·73 (3599·19–4348·87)	3 546 196	3244·21 (2906·40–3457·03)	<0·001
Mortality	189 921	227·23 (196·05–239·23)	213 996	201·40 (176·80–214·06)	172 850	158·13 (141·31–169·43)	<0·001
Haemorrhagic							
Incidence	237 332	283·95 (261·35–306·24)	268 304	252·51 (233·33–273·71)	273 711	250·40 (232·66–270·48)	0·009
MIR	..	0·574 (0·509–0·652)	..	0·492 (0·432–0·575)	..	0·391 (0·346–0·460)	<0·001
DALYs	2 616 799	3130·80 (2838·73–3459·35)	2 547 071	2397·11 (2191·68–2767·00)	2 056 267	1881·16 (1720·23–2175·44)	<0·001
Mortality	136 046	162·77 (146·94–179·98)	131 703	123·95 (113·39–143·70)	106 836	97·74 (89·22–113·97)	<0·001
Aged ≥75 years							
Ischaemic							
Incidence	1 751 254	2824·36 (2627·56–3018·41)	2 031 829	2365·13 (2201·91–2540·09)	2 297 052	2344·00 (2197·01–2503·82)	<0·001
MIR	..	0·537 (0·475–0·584)	..	0·466 (0·428–0·513)	..	0·422 (0·388–0·466)	0·003
DALYs	8 279 171	13 354·53 (11987·70–13857·29)	8 273 750	9660·42 (9191·97–10223·96)	8 231 616	8434·88 (8041·62–9048·47)	<0·001
Mortality	939 894	1511·37 (1353·61–1565·14)	946 346	1082·75 (1031·81–1161·73)	968 866	950·10 (905·53–1030·62)	<0·001
Haemorrhagic							
Incidence	258 372	417·51 (385·93–450·79)	323 137	378·54 (349·89–409·60)	364 687	380·14 (351·37–409·58)	0·035
MIR	..	0·979 (0·882–1·118)	..	0·820 (0·731–0·954)	..	0·748 (0·664–0·870)	<0·001
DALYs	2 359 112	3817·36 (3571·28–4300·03)	2 342 929	2776·87 (2589·80–3203·22)	2 309 063	2457·70 (2273·57–2848·71)	<0·001
Mortality	252 454	407·10 (380·48–462·06)	264 630	306·54 (285·54–353·63)	272 324	275·06 (253·75–320·33)	<0·001
All ages							
Ischaemic							
Incidence	3 392 142	193·02 (179·74–205·88)	3 909 592	172·07 (161·23–183·52)	4 253 257	168·45 (158·25–179·52)	0·001
MIR	..	0·359 (0·316–0·390)	..	0·316 (0·290–0·343)	..	0·285 (0·263–0·311)	<0·001
DALYs	14 914 794	842·32 (749·15–875·30)	15 301 242	671·23 (618·65–703·07)	14 251 741	556·98 (517·25–588·11)	<0·001
Mortality	1 218 033	63·80 (56·45–66·00)	1 235 978	47·77 (45·04–50·41)	1 210 080	40·29 (38·23–43·12)	<0·001
Haemorrhagic							
Incidence	844 105	53·30 (49·44–57·25)	974 336	49·12 (45·75–52·84)	1 050 985	48·81 (45·44–52·13)	0·032
MIR	..	0·652 (0·589–0·729)	..	0·551 (0·492–0·628)	..	0·476 (0·430–0·546)	<0·001
DALYs	10 500 007	695·26 (629·69–753·52)	9 840 594	529·78 (484·51–582·25)	8 569 255	425·13 (385·67–470·63)	<0·001
Mortality	549 858	32·65 (29·95–35·68)	536 700	24·55 (22·67–27·37)	499 809	20·25 (18·57–22·91)	<0·001

**Low-income countries**							

Aged <20 years							
Ischaemic							
Incidence	37 604	1·96 (1·74–2·21)	42 644	1·98 (1·78–2·23)	42 607	1·97 (1·76–2·19)	0·467
MIR		0·158 (0·128–0·196)		0·108 (0·085–0·132)		0·099 (0·078–0·120)	<0·001
DALYs	725 569	37·81 (31·77–44·84)	564 074	26·20 (20·44–31·46)	516 534	23·85 (17·78–28·53)	<0·001
Mortality	8737	0·46 (0·38–0·54)	6788	0·32 (0·25–0·38)	6212	0·29 (0·21–0·34)	<0·001
Haemorrhagic							
Incidence	24 132	1·26 (1·12–1·42)	31 290	1·45 (1·31–1·63)	31 333	1·45 (1·30–1·60)	0·036
MIR		1·153 (0·858–1·439)		0·695 (0·484–0·895)		0·638 (0·433–0·832)	<0·001
DALYs	3 842 839	200·23 (148·70–247·29)	2909 492	135·12 (94·33–166·46)	2658 426	122·75 (84·26–154·03)	<0·001
Mortality	47 489	2·47 (1·84–3·05)	36 251	1·68 (1·18–2·08)	33 152	1·53 (1·05–1·91)	<0·001
Aged ≥20–64 years							
Ischaemic							
Incidence	1 048 665	52·22 (46·05–59·19)	1 605 620	57·20 (50·38–65·30)	1 958 154	62·80 (55·35–71·12)	0·023
MIR	..	0·162 (0·125–0·229)	..	0·131 (0·105–0·176)	..	0·116 (0·096–0·152)	0·001
DALYs	5 724 385	285·06 (235·39–382·78)	7 242 596	258·01 (221·90–331·52)	7 822 374	250·88 (222·65–313·29)	0·013
Mortality	169 222	8·43 (6·79–11·60)	209 449	7·46 (6·28–9·87)	226 360	7·26 (6·36–9·35)	0·006
Haemorrhagic							
Incidence	932 934	46·46 (40·34–53·15)	1 699 290	60·54 (52·05–71·01)	2 048 034	65·68 (56·36–76·71)	0·001
MIR	..	0·736 (0·569–0·918)	..	0·494 (0·397–0·602)	..	0·422 (0·333–0·515)	<0·001
DALYs	23 320 724	1161·33 (946·76–1394·12)	28 837 888	1027·33 (877·98–1183·65)	29 450 146	944·51 (802·88–1095·88)	<0·001
Mortality	682 773	34·00 (27·64–41·04)	833 453	29·69 (25·27–34·26)	858 841	27·54 (23·33–32·06)	<0·001
Aged 65–74 years							
Ischaemic							
Incidence	1 448 192	1152·07 (993·10–1330·23)	2 242 233	1178·40 (1023·70–1368·17)	2 503 522	1197·93 (1046·63–1376·61)	0·363
MIR	..	0·188 (0·143–0·261)	..	0·167 (0·132–0·222)	..	0·152 (0·125–0·200)	0·039
DALYs	5 256 373	4181·55 (3367·95–5547·71)	7 286 998	3829·67 (3233·25–4924·07)	7 455 071	3567·24 (3120·81–4518·74)	0·005
Mortality	270 306	215·03 (171·34–289·19)	372 174	195·60 (163·42–253·47)	379 837	181·75 (157·63–232·97)	0·006
Haemorrhagic							
Incidence	635 720	505·73 (426·21–595·26)	1 138 008	598·08 (507·06–706·31)	1 243 472	595·00 (507·58–703·97)	0·085
MIR	..	0·868 (0·648–1·106)	..	0·632 (0·498–0·791)	..	0·563 (0·434–0·700)	<0·001
DALYs	10 345 455	8230·02 (6 557·97–10 024·44)	13 489 314	7089·29 (5966·41–8334·24)	13 110 681	6273·45 (5331·21–7356·20)	<0·001
Mortality	547 366	435·44 (345·66–530·97)	714 434	375·47 (315·28–442·45)	695 399	332·75 (282·67–390·97)	<0·001
Aged ≥75 years							
Ischaemic							
Incidence	1 312 155	2367·54 (2026·74–2735·51)	2 297 208	2537·52 (2202·95–2941·36)	2 811 999	2575·40 (2240·67–2950·24)	0·222
MIR	..	0·440 (0·355–0·562)	..	0·384 (0·312–0·480)	..	0·362 (0·298–0·443)	0·046
DALYs	5 507 099	9938·45 (8486·20–12 399·96)	8 176 998	9013·12 (7860·57–10 865·64)	9 343 686	8553·44 (7572·78–10 099·22)	0·001
Mortality	574 779	1075·73 (915·74–1336·49)	877 484	997·48 (870·23–1204·49)	1 012 930	949·88 (838·56–1128·36)	0·01
Haemorrhagic							
Incidence	403 286	713·83 (603·31–847·38)	793 903	861·85 (735·11–1020·98)	951 173	859·36 (729·17–1012·58)	0·065
MIR	..	1·479 (1·090–1·900)	..	1·094 (0·855–1·356)	..	1·008 (0·784–1·250)	0·002
DALYs	5 873 138	10 249·10 (7846·57–12 615·18)	8 302 502	8907·91 (7406·47–10 533·99)	9 054 390	8113·74 (6818·88–9519·69)	0·002
Mortality	591 886	1072·90 (819·30–1329·49)	862 258	955·71 (789·22–1138·04)	951 562	874·84 (736·84–1026·62)	0·007
All ages							
Ischaemic							
Incidence	3 846 616	170·53 (148·24–195·28)	6 187 705	178·68 (156·28–205·59)	7 316 281	181·70 (159·10–206·78)	0·267
MIR	..	0·266 (0·213–0·354)	..	0·238 (0·193–0·303)	..	0·223 (0·186–0·276)	0·051
DALYs	17 213 426	734·86 (619·49–948·36)	23 270 664	658·32 (571·81–818·09)	25 137 666	613·93 (550·41–748·03)	<0·001
Mortality	1 023 044	50·13 (42·02–64·07)	1 465 895	45·77 (39·69–56·27)	1 625 339	43·05 (38·25–51·96)	<0·001
Haemorrhagic							
Incidence	1 996 072	81·40 (69·54–94·31)	3 662 492	98·80 (84·77–115·66)	4 274 013	99·43 (85·37–116·28)	0·040
MIR	..	0·932 (0·708–1·177)	..	0·668 (0·534–0·822)	..	0·595 (0·470–0·729)	<0·001
DALYs	43 382 156	1614·23 (1292·98–1946·36)	53 539 196	1363·83 (1154·83–1580·73)	54 273 644	1207·21 (1024·82–1408·04)	<0·001
Mortality	1 869 514	80·37 (63·72–96·98)	2 446 397	69·29 (58·11–81·26)	2 538 954	61·93 (52·53–72·34)	<0·001

**Globally**							

Aged <20 years							
Ischaemic							
Incidence	45 872	2·03 (1·85–2·25)	49 324	2·01 (1·83–2·23)	48 716	1·98 (1·80–2·18)	0·371
MIR	..	0·136 (0·112–0·165)	..	0·098 (0·077–0·118)	..	0·090 (0·070–0·108)	<0·001
DALYs	760 596	33·73 (28·57–39·77)	592 518	24·13 (18·80–28·77)	539 446	21·96 (16·39–26·10)	<0·001
Mortality	9121	0·40 (0·34–0·48)	7079	0·29 (0·22–0·34)	6435	0·26 (0·20–0·31)	<0·001
Haemorrhagic							
Incidence	29 240	1·30 (1·17–1·43)	35 025	1·43 (1·29–1·58)	34 727	1·41 (1·29–1·56)	0·120
MIR	..	1·001 (0·765–1·237)	..	0·642 (0·452–0·819)	..	0·592 (0·404–0·762)	<0·001
DALYs	3 997 346	177·24 (132·48–217·62)	2 981 205	121·43 (85·31–149·09)	2 713 752	110·50 (76·32–138·09)	<0·001
Mortality	49 464	2·19 (1·64–2·68)	37 159	1·51 (1·07–1·86)	33 843	1·38 (0·95–1·72)	<0·001
Aged ≥20–64 years							
Ischaemic							
Incidence	1 667 786	61·35 (56·39–66·65)	2 268 891	63·34 (57·92–69·84)	2 701 367	69·09 (63·00–76·01)	0·035
MIR	..	0·154 (0·133–0·194)	..	0·126 (0·108–0·159)	..	0·109 (0·095–0·134)	<0·001
DALYs	8 543 219	314·27 (278·37–384·70)	9 892 888	276·19 (249·70–334·81)	10 273 392	262·73 (241·48–312·86)	<0·001
Mortality	257 056	9·46 (8·30–11·77)	284 793	7·95 (7·09–9·86)	294 500	7·53 (6·85–9·24)	<0·001
Haemorrhagic							
Incidence	1 276 228	46·95 (42·30–51·87)	2 078 450	58·03 (51·33–66·39)	2 457 227	62·84 (55·30–71·67)	0·001
MIR	..	0·662 (0·544–0·787)	..	0·470 (0·389–0·557)	..	0·400 (0·325–0·478)	<0·001
DALYs	28 690 314	1055·41 (895·45–1232·70)	33 716 770	941·29 (818·42–1072·58)	33 598 744	859·26 (741·90–985·04)	<0·001
Mortality	842 156	30·98 (26·27–36·36)	972 913	27·16 (23·57–31·04)	978 799	25·03 (21·57–28·77)	<0·001
Aged 65–74 years							
Ischaemic							
Incidence	2 461 690	1176·23 (1073·89–1291·17)	3 450 045	1163·46 (1053·37–1287·36)	3 710 404	1165·71 (1057·96–1284·45)	0·426
MIR	..	0·187 (0·161–0·229)	..	0·170 (0·147–0·205)	..	0·149 (0·130–0·180)	0·001
DALYs	9 038 136	4318·55 (3864·39–5068·30)	11 635 754	3923·93 (3559·88–4600·94)	11 001 267	3456·31 (3184·54–4085·80)	<0·001
Mortality	460 226	219·90 (195·49–261·22)	586 170	197·67 (178·67–234·07)	552 687	173·64 (158·88–207·65)	<0·001
Haemorrhagic							
Incidence	873 052	417·16 (369·14–471·98)	1 406 313	474·25 (414·57–545·31)	1 517 183	476·66 (418·82–549·38)	0·078
MIR	..	0·786 (0·627–0·967)	..	0·605 (0·491–0·730)	..	0·531 (0·426–0·640)	<0·001
DALYs	12 962 254	6193·55 (5168·67–7330·20)	16 036 385	5407·96 (4672·18–6260·19)	15 166 948	4765·06 (4129·38–5527·14)	<0·001
Mortality	683 412	326·54 (272·05–386·99)	846 138	285·34 (245·88–331·99)	802 234	252·04 (218·06–292·88)	<0·001
Aged ≥75 years							
Ischaemic							
Incidence	3 063 410	2614·89 (2426·49–2809·55)	4 329 037	2452·72 (2245·04–2674·44)	5 109 051	2472·93 (2279·15–2687·39)	0·176
MIR	..	0·495 (0·450–0·547)		0·422 (0·379–0·483)		0·389 (0·348–0·450)	<0·001
DALYs	13 786 270	11 766·13 (11 034·98–12 688·85)	16 450 747	9314·39 (8699·15–10 498·56)	17 575 302	8509·08 (7979·52–9562·85)	<0·001
Mortality	1 514 673	1313·55 (1225·05–1407·29)	1 823 831	1040·14 (972·42–1184·36)	1 981 797	952·73 (893·26–1082·61)	<0·001
Haemorrhagic							
Incidence	661 658	558·61 (503·36–624·07)	1 117 040	630·25 (558·49–712·17)	1 315 860	640·06 (569·10–724·72)	0·046
MIR	..	1·280 (1·041–1·542)		1·012 (0·841–1·208)		0·934 (0·767–1·107)	0·001
DALYs	8 232 250	6899·22 (5819·05–8101·89)	10 645 431	5985·97 (5185·38–6900·83)	11 363 453	5544·55 (4848·87–6377·47)	<0·001
Mortality	844 340	719·66 (605·91–844·83)	1 126 888	638·52 (554·36–736·04)	1 223 886	592·56 (517·87–681·36)	<0·001
All ages							
Ischaemic							
Incidence	7 238 758	181·19 (167·30–196·23)	10 097 297	175·46 (160·08–192·26)	11 569 538	176·44 (161·46–192·21)	0·324
MIR	..	0·310 (0·278–0·352)		0·268 (0·237–0·310)		0·245 (0·219–0·285)	<0·001
DALYs	32 128 220	795·80 (733·54–906·40)	38 571 908	667·77 (617·11–774·20)	39 389 408	597·80 (559·75–691·68)	<0·001
Mortality	2 241 077	57·59 (53·69–63·97)	2 701 873	46·92 (43·58–53·67)	2 835 419	42·27 (39·60–48·71)	<0·001
Haemorrhagic							
Incidence	2 840 177	69·36 (62·46–77·18)	4 636 828	80·33 (71·40–91·69)	5 324 997	81·52 (72·27–92·82)	0·033
MIR	..	0·847 (0·692–1·009)		0·643 (0·536–0·766)		0·571 (0·471–0·676)	<0·001
DALYs	53 882 164	1266·94 (1068·41–1484·26)	63 379 792	1081·81 (935·41–1234·23)	62 842 896	956·22 (827·57–1104·44)	<0·001
Mortality	2 419 372	59·66 (50·61–69·71)	2 983 097	51·61 (44·68–59·07)	3 038 763	46·14 (40·13–53·15)	<0·001

Data are point estimates (95% CIs), unless otherwise indicated. DALYs=disability-adjusted life-years.

* p-values are for the trend in rates between 1990 and 2010 only.

**Table 4:** Mean age of incident and fatal strokes in 1990, 2005, and 2010, by stroke type and country income level

	High-income				Low-income and middle-income				Globally			
	1990	2005	2010	p value*	1990	2005	2010	p value*	1990	2005	2010	p value*
**Ischaemic**												

Incidence	75·4 (0·13)	75·9 (0·13)	76·2 (0·13)	<0·001	69·6 (0·12)	70·6 (0·11)	70·8 (0·12)	<0·001	72·9 (0·11)	73·0 (0·10)	73·1 (0·10)	0·079
Mortality	80·7 (0·12)	81·3 (0·22)	82·7 (0·21)	<0·001	75·1 (0·32)	76·4 (0·27)	77·1 (0·26)	<0·001	78·1 (0·27)	78·6 (0·20)	79·4 (0·19)	<0·001

**Haemorrhagic**												

Incidence	67·6 (0·13)	68·7 (0·14)	69·1 (0·15)	<0·001	62·8 (0·15)	63·7 (0·13)	63·8 (0·13)	<0·001	64·7 (0·11)	65·0 (0·10)	65·1 (0·11)	0·001
Mortality	71·6 (0·29)	73·0 (0·30)	74·8 (0·32)	<0·001	66·8 (0·29)	68·2 (0·28)	68·9 (0·31)	<0·001	68·0 (0·23)	69·1 (0·25)	69·9 (0·30)	<0·001

Data are mean (SD).

* p-values are for the trend in mean age between 1990 and 2010 only

## Panel: Research in context

### Systematic review

We searched Medline, Embase, LILACS, Scopus, PubMed, Science Direct, Global Health Database, the WHO library, and WHO regional databases between 1990 and 2010 to identify studies published between 1990 and 2010. We searched the databases with various key or title words: “stroke”, “isch(a)emic stroke”, “intracerebral”, “intraparenchymal”, “subarachnoid”, “h(a)emorrhage” AND title or key-words “population-based”, “communitybased”, “community”, “epidemiology”, “epidemiological”, “incidence”, “attack rates”, “survey”, “surveillance”, “mortality”, “morbidity”, “fatality”, “case-fatality”, or “trends”. We assessed pathological types of stroke only for studies that had findings for head CT, MRI, or brain autopsy findings available for at least 70% of stroke cases. We classified cases of stroke with no neuroimaging or autopsy verification of pathological type of the stroke as a stroke of undetermined pathological type. We categorised pathological types of stroke into two groups: ischaemic strokes and haemorrhagic strokes (intracerebral haemorrhage and subarachnoid haemorrhage combined). We assessed only first-ever stroke events.

### Interpretation

This study is the first to report the global burden of ischaemic and haemorrhagic stroke in terms of incidence, mortality, DALYs lost, and mortality-to-incidence ratio across GBD regions and countries in 1990, 2005, and 2010, and across all age groups of the population. Our results show that age-standardised mortality rates for ischaemic and haemorrhagic stroke have decreased in the past two decades; however, the absolute number of people who have these stroke types every year, and the number with related deaths and DALYs lost, is increasing, with most of the burden in low-income and middle-income countries. Our findings could be used as a vital source of information for future planning of preventive strategies for stroke, but further study is needed to identify which subgroups of the population are at greatest risk and who will benefit most from targeted preventive efforts.
